# Supplementary material for: CPTCs Drive Somatic‐Visceral Communication via the Wnt Axis in Somatic Mechanotherapy: A Single‐Cell Deep Learning Study
Source: FASEB J. 2026 Aug 8;40(15):e72176. doi: 10.1096/fj.202600755RRR (PMC13452619; doi:10.1096/fj.202600755RRR)
Supplement: Supplementary file 1 [file FSB2-40-e72176-s001.docx]

**Table S1. Mechanospecific Differentially Expressed Genes in Fascial CPTCs Following Acupuncture**

| **gene** | **avg_log2FC** | **p_val_adj** | **gene_set** |
| --- | --- | --- | --- |
| Hspa1b | 1.21230530395239 | 5.00076198288841e-179 | Mechanoresponsive |
| Hspa8 | 0.494622746115392 | 4.56074060356613e-117 | Mechanoresponsive |
| Jun | 0.760667330533413 | 6.17907294505368e-92 | Mechanoresponsive |
| Fos | 0.855731493728587 | 1.71429484490759e-64 | Mechanoresponsive |
| Hspa1a | 0.185360255640885 | 1.48409066008886e-46 | Mechanoresponsive |
| Atf3 | 0.616919424998338 | 1.65223726617584e-34 | Mechanoresponsive |
| Hsp90aa1 | 0.470726172581825 | 1.9701868092712e-33 | Mechanoresponsive |
| Egr1 | 0.387359034034171 | 3.43717452992231e-24 | Mechanoresponsive |
| Fosb | 0.369009959207871 | 9.53178685173476e-17 | Mechanoresponsive |
| Hsp90ab1 | 0.140353727117842 | 5.48286191755859e-12 | Mechanoresponsive |
| Egr2 | 0.101166713106644 | 9.95763296691295e-12 | Mechanoresponsive |
| Piezo2 | 0.0342887251291897 | 5.99796012364753e-06 | Mechanoresponsive |
| Stk4 | 0.0263701591621472 | 0.146288495114636 | Mechanoresponsive |
| Trpv4 | 0.0204872154403918 | 0.211867894605373 | Mechanoresponsive |
| Lats1 | -0.059704696457363 | 1 | Mechanoresponsive |
| Itgb1 | -0.0410069296992925 | 1 | Mechanoresponsive |
| Stk3 | -0.0184319022801447 | 1 | Mechanoresponsive |
| Piezo1 | -0.0116072135949198 | 1 | Mechanoresponsive |
| Wwtr1 | -0.0260995683132633 | 1 | Mechanoresponsive |
| Vcl | -0.0568145740499281 | 1 | Mechanoresponsive |
| Actn4 | -0.025945660999106 | 1 | Mechanoresponsive |
| Trpv2 | -0.0043453094986729 | 1 | Mechanoresponsive |
| Yap1 | -0.0157076572930078 | 1 | Mechanoresponsive |
| Lats2 | -0.0246498594089684 | 1 | Mechanoresponsive |
| Itga5 | -0.014639830708952 | 1 | Mechanoresponsive |
| Trpc1 | -0.00655937799109833 | 1 | Mechanoresponsive |
| Actn1 | 0.00600316767068746 | 1 | Mechanoresponsive |
| Ccl4 | -0.455782071712733 | 4.47253950922398e-135 | Inflammatory |
| Il6 | 0.638536729368017 | 6.28638178333621e-38 | Inflammatory |
| Nfkb1 | -0.429090610767849 | 1.78095899778719e-35 | Inflammatory |
| Cxcl2 | -0.206915891464816 | 8.65771556053889e-24 | Inflammatory |
| Ccl3 | -0.0549010974202764 | 2.38835233814861e-06 | Inflammatory |
| Cxcl1 | 0.301224372286183 | 0.000359761533894365 | Inflammatory |
| Rel | -0.116818274391292 | 0.00386774634042724 | Inflammatory |
| Casp1 | 0.0249894557053892 | 0.666057405512876 | Inflammatory |
| Traf6 | 0.0115934378083243 | 1 | Inflammatory |
| Nfkbib | -0.0547332929318085 | 1 | Inflammatory |
| Il10 | -0.0232781189644178 | 1 | Inflammatory |
| Irak4 | 0.0161109025438979 | 1 | Inflammatory |
| Cxcl10 | 0.251020899467879 | 1 | Inflammatory |
| Nfkb2 | -0.0466622435765217 | 1 | Inflammatory |
| Aim2 | 0.00271426035801003 | 1 | Inflammatory |
| Il18 | -0.0129942690002788 | 1 | Inflammatory |
| Ifng | 0.000249262256598771 | 1 | Inflammatory |
| Tlr4 | -0.0121335554139806 | 1 | Inflammatory |
| Rela | -0.0294566000406148 | 1 | Inflammatory |
| Il1b | -0.00857200842966581 | 1 | Inflammatory |
| Ccl2 | 0.0415894482929717 | 1 | Inflammatory |
| Relb | -0.0291848366260134 | 1 | Inflammatory |
| Nlrp3 | 4.15886466269885e-06 | 1 | Inflammatory |
| Tnf | -0.0118925334528178 | 1 | Inflammatory |
| Nfkbia | 0.0325739478636184 | 1 | Inflammatory |
| Tlr2 | -0.0259027449293151 | 1 | Inflammatory |
| Irak1 | -0.0146636528164847 | 1 | Inflammatory |
| Myd88 | -0.0241830970178367 | 1 | Inflammatory |
| Il4 | 0 | 1 | Inflammatory |

*Note: avg_log2FC, average log2 fold-change; p_val_adj, Bonferroni-adjusted p-value; gene_set, functional annotation (Inflammatory, Mechanoresponsive).*
