# Supplementary material for: CPTCs Drive Somatic‐Visceral Communication via the Wnt Axis in Somatic Mechanotherapy: A Single‐Cell Deep Learning Study
Source: FASEB J. 2026 Aug 8;40(15):e72176. doi: 10.1096/fj.202600755RRR (PMC13452619; doi:10.1096/fj.202600755RRR)
Supplement: Supplementary file 2 [file FSB2-40-e72176-s003.docx]

**Table S3. SHAP Feature Attribution Scores for All Genes in the Cross-Study CPTC Model**

|  | **gene** | **shap** |
| --- | --- | --- |
| 1463 | Hspa1b | 0.6165131776284847 |
| 610 | Csn2 | 0.603208837143303 |
| 95 | ENSRNOG00000050647 | 0.5118628300732707 |
| 217 | Csn1s1 | 0.43305170407838367 |
| 298 | Plau | 0.38464894155997525 |
| 66 | Ptgs2 | 0.3640009441158213 |
| 144 | Mrgprg | 0.3487151601658021 |
| 251 | Lum | 0.33058816249524414 |
| 840 | Il1r2 | 0.325628919095414 |
| 630 | Glycam1 | 0.30360086123470303 |
| 296 | C6 | 0.27297646270108356 |
| 105 | Sfrp4 | 0.2693965010770887 |
| 335 | Csn3 | 0.2570192937774473 |
| 1244 | RT1-T24-4 | 0.2512501591143813 |
| 1697 | Galnt15 | 0.24549315668612331 |
| 81 | Il6 | 0.2332610764603536 |
| 920 | Art3 | 0.23288153879072337 |
| 87 | Cxcl1 | 0.22907266317390346 |
| 0 | S100a9 | 0.2147522439444286 |
| 304 | Inmt | 0.2141535877217057 |
| 167 | Cxcl13 | 0.21019454710337457 |
| 359 | Ccn5 | 0.20801840315658737 |
| 2446 | Eno2 | 0.20606610042733198 |
| 238 | Ccn1 | 0.20427656107308706 |
| 418 | Tnfrsf11b | 0.19780564364808773 |
| 435 | Cfh | 0.18891747613086193 |
| 700 | Fosb | 0.18669444292090237 |
| 947 | Cd9 | 0.1859156246209131 |
| 714 | Aldh1a3 | 0.18574196295607015 |
| 1809 | Sult1a1 | 0.18108469421830914 |
| 3115 | S100a6 | 0.1802643173767733 |
| 1042 | Mfap2 | 0.17341743432714182 |
| 1189 | ENSRNOG00000069024 | 0.16653742380915806 |
| 1078 | Tppp3 | 0.16304670596754045 |
| 1991 | C1qtnf2 | 0.16236195751762406 |
| 2193 | C1qtnf7 | 0.1603455035000963 |
| 453 | Cxcl12 | 0.1576617174737988 |
| 933 | Anxa3 | 0.1571624737268566 |
| 1446 | Thbs2 | 0.15672520359082306 |
| 2651 | Myh10 | 0.15547383272324133 |
| 209 | Mfap4 | 0.15269073204070688 |
| 203 | Ccl2 | 0.1520263799588148 |
| 2606 | Tmem140 | 0.15165226390866351 |
| 744 | Id3 | 0.15058462451187765 |
| 248 | Cfd | 0.14815451492903892 |
| 279 | C1qb | 0.14556971334529237 |
| 764 | Jun | 0.1453292395694356 |
| 230 | Cd74 | 0.14503885556341944 |
| 1306 | Btg1 | 0.14432250635279192 |
| 522 | Ndrg2 | 0.14415890960567176 |
| 1351 | Plpp3 | 0.14354911124353986 |
| 289 | Sparcl1 | 0.14337531347647664 |
| 349 | Sdc4 | 0.14080709286718626 |
| 2748 | Camk2n1 | 0.14039393751598642 |
| 1256 | Ftl1 | 0.14009132196151103 |
| 973 | Gap43 | 0.1387020716240794 |
| 2832 | ENSRNOG00000071035 | 0.13752326434753664 |
| 2197 | Ebf2 | 0.1374022479803974 |
| 1600 | Fibin | 0.13633929090260005 |
| 721 | Wap | 0.13507918107657046 |
| 1833 | Aif1l | 0.13458789129541646 |
| 2428 | ENSRNOG00000031041 | 0.13441330978816246 |
| 910 | Procr | 0.1335016827368808 |
| 1699 | Ace | 0.13300448765144723 |
| 1936 | Gnpnat1 | 0.1325998099866159 |
| 3016 | Prr27 | 0.12972482858165804 |
| 960 | Efemp1 | 0.127971942230569 |
| 917 | Csrp2 | 0.12740168023746395 |
| 1621 | Engase | 0.1255658450817338 |
| 1819 | Ndrg1 | 0.1254649422171506 |
| 2515 | Adgrg2 | 0.12543263908139934 |
| 191 | Has1 | 0.12517797528035557 |
| 376 | Steap4 | 0.12502859020117651 |
| 673 | Ifrd1 | 0.1249636848568942 |
| 670 | Sgk1 | 0.12481848882162666 |
| 3161 | Klhl21 | 0.12455547741970716 |
| 1368 | Plat | 0.12299453339795367 |
| 479 | Errfi1 | 0.1227764890617857 |
| 310 | Itm2a | 0.12251499406270941 |
| 506 | Dact2 | 0.12225810527057177 |
| 2594 | Zfp385a | 0.12148146771476542 |
| 609 | Gpx3 | 0.12115842286399148 |
| 1691 | Pcdh20 | 0.1208187398088835 |
| 2390 | Slco3a1 | 0.12079520854896815 |
| 2348 | Glul | 0.12079382669947 |
| 2794 | Mitf | 0.11982735010415718 |
| 688 | Coq10b | 0.11898782753657322 |
| 833 | Fgl2 | 0.1169282753102227 |
| 837 | Fos | 0.11648256350037184 |
| 1958 | Plpp1 | 0.11409273294645683 |
| 187 | Plac8 | 0.11356634783879489 |
| 477 | Ier3 | 0.11166876893662417 |
| 2223 | Gbp6.1 | 0.11132885322212457 |
| 1327 | Tnfrsf12a | 0.1109833076081142 |
| 3063 | Limch1 | 0.11038209852940642 |
| 1438 | Klf9 | 0.10956531757080476 |
| 1570 | Enpp3 | 0.10951217044536864 |
| 232 | Gadd45a | 0.10944570889885877 |
| 137 | Eln | 0.1088685125768829 |
| 2563 | Slc29a1 | 0.10882708847219033 |
| 2778 | Sema6a | 0.10709305598366829 |
| 355 | Filip1l | 0.1069787059197747 |
| 637 | Postn | 0.10614342522764067 |
| 1520 | Ecm1 | 0.10582377796859933 |
| 77 | AABR07060872.1 | 0.10489470841785341 |
| 893 | Kitlg | 0.10460708076318045 |
| 814 | C4a | 0.10448986311071247 |
| 1672 | Omd | 0.10436784905160941 |
| 691 | Enc1 | 0.10355504144380412 |
| 2489 | Pdlim4 | 0.10345449981232833 |
| 1335 | ENSRNOG00000019926 | 0.10283961821245265 |
| 1517 | Cpz | 0.10267592464287646 |
| 1289 | Akap12 | 0.10254860648416413 |
| 3132 | Slc43a1 | 0.10177960653316201 |
| 2261 | Abcc9 | 0.10139523400137451 |
| 600 | Nr4a1 | 0.10128636755568152 |
| 731 | ENSRNOG00000004712 | 0.10110252562347585 |
| 1377 | Tmem178a | 0.10038203202828992 |
| 2268 | Pcdh7 | 0.09983679819750808 |
| 1192 | Plagl1 | 0.09939581815233088 |
| 961 | Tyrobp | 0.09879106184682443 |
| 2270 | Spsb1 | 0.09838021788836038 |
| 1807 | Heg1 | 0.09824411432315938 |
| 2175 | Lrrc17 | 0.09812115106803589 |
| 1799 | Bmp7 | 0.09791623363716923 |
| 823 | Ccnd1 | 0.09784554522716786 |
| 2294 | Dlc1 | 0.09703929829238955 |
| 706 | Akr1c14 | 0.09701435785839518 |
| 1771 | Pkig | 0.09697469915257476 |
| 813 | Cebpd | 0.09692775302459047 |
| 11 | Ccl4 | 0.09614674271182048 |
| 1856 | Fbln1 | 0.09580641303542031 |
| 923 | S100a4 | 0.09579718682030733 |
| 2093 | Arhgap29 | 0.09545005957758013 |
| 1418 | Vps25 | 0.09539391969929598 |
| 2192 | Agtr1a | 0.09484822601844944 |
| 1885 | Msx1 | 0.09462094405740523 |
| 1651 | Tek | 0.09444350784894609 |
| 123 | Cxcl2 | 0.09407313911258447 |
| 2456 | Clic2 | 0.09401803659781877 |
| 2149 | Tspan11 | 0.09377249779085314 |
| 2378 | Ar | 0.09312982635901895 |
| 1401 | Tgfb2 | 0.09267092145182487 |
| 2099 | Ndn | 0.09247925293128653 |
| 1967 | Phlda3 | 0.09208877623174294 |
| 1953 | Steap1 | 0.09187990687763821 |
| 2844 | Cd34 | 0.09151846399212314 |
| 221 | Wfdc1 | 0.09137070830443438 |
| 2301 | Hlf | 0.09076641305377638 |
| 1416 | Loxl1 | 0.09048647837938821 |
| 1179 | Akr1b8 | 0.08982202920829382 |
| 1409 | Vegfa | 0.08975435299115289 |
| 1547 | Stmn4 | 0.08960018453170618 |
| 952 | Adora2b | 0.08945419473874543 |
| 2047 | Tmeff2 | 0.08929608490800492 |
| 1889 | Lnx1 | 0.08925172281247891 |
| 593 | Gpnmb | 0.089157030365481 |
| 2034 | Tspan7 | 0.0891448187341261 |
| 2737 | Srgap1 | 0.0890501383861261 |
| 2383 | Bcl3 | 0.08904353805524892 |
| 280 | C1qa | 0.08882710393496865 |
| 831 | Klf6 | 0.08870729745925879 |
| 835 | Ifit2 | 0.08869222480676751 |
| 332 | C3 | 0.08849340088149966 |
| 604 | Rpgr | 0.08780620498724465 |
| 2311 | Rcan2 | 0.08780563023620051 |
| 1146 | Col18a1 | 0.08778858574234734 |
| 2636 | Bcat1 | 0.08758282339534171 |
| 297 | Hes1 | 0.08737594565654547 |
| 1123 | Lurap1l | 0.08722043203221347 |
| 293 | Pf4 | 0.08701614558357473 |
| 1371 | Pthlh | 0.0870054713231676 |
| 1260 | ENSRNOG00000069594 | 0.08699219042144365 |
| 1523 | Ptgs1 | 0.08673089389222596 |
| 752 | Fbn1 | 0.08642200907583836 |
| 283 | ENSRNOG00000061379 | 0.08570553137737508 |
| 1796 | Gstm3 | 0.08566808184617772 |
| 148 | Hmox1 | 0.085060210088539 |
| 1522 | Clec3b | 0.08496580471118084 |
| 1606 | Ier5l | 0.08445890399503483 |
| 2357 | Creb3l1 | 0.08434689825163143 |
| 2020 | Slc7a6os | 0.08382655421157706 |
| 676 | Svep1 | 0.08344746617427774 |
| 1528 | Arl4a | 0.08343609525760123 |
| 2269 | Itga11 | 0.08307577776227965 |
| 1832 | Selenbp1 | 0.08295668466579305 |
| 1361 | Arg1 | 0.08282592386000123 |
| 316 | ENSRNOG00000049075 | 0.0825606394839043 |
| 1574 | RT1-T24-1 | 0.08213224171325334 |
| 1119 | Zfp36 | 0.08194109370154318 |
| 2318 | Nudt4 | 0.08189876465225493 |
| 2204 | Cycs | 0.08185869097354295 |
| 1703 | Prdm8 | 0.08182818165937088 |
| 2841 | Trib3 | 0.08177694468131155 |
| 2550 | Lamc1 | 0.08121983561934627 |
| 2974 | Lpar1 | 0.0811395224408386 |
| 976 | Pdlim1 | 0.08112139362550996 |
| 2809 | ENSRNOG00000003715 | 0.08107470819889498 |
| 1341 | ENSRNOG00000068238 | 0.08098763043263119 |
| 2164 | ENSRNOG00000039496 | 0.08097760817277219 |
| 2123 | Olfml3 | 0.08082365701255452 |
| 757 | Vcam1 | 0.08031779911425291 |
| 1283 | Crem | 0.08013802636758832 |
| 996 | Lbp | 0.07925650530075956 |
| 2052 | Lamb1 | 0.07847636149201734 |
| 597 | Gm2a | 0.07844117384081321 |
| 2296 | Gpx7 | 0.07818085722218379 |
| 2702 | Cdk5rap2 | 0.07809478148244395 |
| 2066 | Rin2 | 0.0774658097940032 |
| 584 | Hsph1 | 0.07730595800422291 |
| 1007 | Cd55 | 0.07721370308570338 |
| 1488 | Plekhf1 | 0.07707234066383237 |
| 2686 | Lmo4 | 0.07705485026505132 |
| 1025 | Slit3 | 0.07700993414140495 |
| 948 | Oas1a | 0.07684292507964113 |
| 3151 | Serinc5 | 0.07677563013214031 |
| 1185 | Clec2l | 0.07676105060824603 |
| 1979 | Dnaja1 | 0.0765504829706625 |
| 804 | Spry2 | 0.07612095461054416 |
| 2903 | Ehbp1 | 0.07593484291483707 |
| 1934 | Bst2 | 0.0756203523254584 |
| 1440 | Jag1 | 0.07536647507844624 |
| 1 | S100a8 | 0.0753411732902976 |
| 2299 | Sapcd1 | 0.07516695563003103 |
| 125 | Tpm1 | 0.07485073868250136 |
| 2259 | Pkdcc | 0.0746681133182521 |
| 1457 | ENSRNOG00000004160 | 0.07442533953794353 |
| 1210 | Irf7 | 0.07420396282753211 |
| 2512 | Lgals9 | 0.07418613866827023 |
| 1968 | Pdp2 | 0.073987343591114 |
| 2016 | ENSRNOG00000018951 | 0.07367734285469124 |
| 2484 | Tubb5 | 0.07334296763830273 |
| 1589 | Ppl | 0.07317165442514555 |
| 711 | Btg2 | 0.0726478150519685 |
| 1445 | Pxdn | 0.07228610397215211 |
| 2356 | Lrrc32 | 0.07165296456893797 |
| 1931 | Creb5 | 0.07142160081789353 |
| 871 | Nfil3 | 0.07122397063294958 |
| 2377 | Clmp | 0.07119554775696593 |
| 570 | Clec10a | 0.07115664394752097 |
| 1623 | Adamts5 | 0.07080111700563094 |
| 878 | Irf1 | 0.07074142836053955 |
| 130 | RGD1564664 | 0.07053509588717909 |
| 2690 | Ncoa7 | 0.07025033955616458 |
| 208 | Id4 | 0.07003656746633735 |
| 938 | Cst3 | 0.06975016214935517 |
| 18 | G0s2 | 0.06933208026940588 |
| 1963 | Rasl10a | 0.06928174928069059 |
| 382 | Atf3 | 0.06924327398113653 |
| 2075 | Duox1 | 0.06915822795401401 |
| 892 | ENSRNOG00000014464 | 0.06886468166687182 |
| 617 | Egr1 | 0.068835363021821 |
| 2637 | Nnmt | 0.06883326207630067 |
| 2331 | Blvra | 0.06861083324679466 |
| 3015 | Ngf | 0.06837826847958663 |
| 464 | Ifitm1 | 0.0683605816378788 |
| 1454 | ENSRNOG00000064648 | 0.06810617655883064 |
| 1750 | Sned1 | 0.06808537230710827 |
| 613 | Ifi27l2b | 0.06770394470640237 |
| 761 | Akr1c15 | 0.06757330442961951 |
| 273 | C1qc | 0.06715659393183118 |
| 699 | Dhrs7 | 0.0671437388178727 |
| 1783 | Adamts12 | 0.06711971588085819 |
| 1034 | AABR07028488.1 | 0.06653604673336642 |
| 1696 | ENSRNOG00000000875 | 0.0663584564154382 |
| 857 | Sod2 | 0.0662236669167576 |
| 2336 | Zc3hav1 | 0.06617237616368535 |
| 541 | Ntrk2 | 0.0655268526136247 |
| 1439 | ENSRNOG00000065519 | 0.06532796011819039 |
| 724 | Bmp2 | 0.06527229073163011 |
| 1028 | P3h3 | 0.0651376010587614 |
| 991 | Olfml2a | 0.06463231244323493 |
| 2459 | Fzd2 | 0.06457433761549963 |
| 862 | Gem | 0.06456959963367728 |
| 1001 | Gpx1 | 0.06450858144366266 |
| 2883 | Gstm1 | 0.06442004824068179 |
| 1734 | Mmp14 | 0.06404177986865515 |
| 844 | Tuba1c | 0.064024172747533 |
| 194 | Abca8a | 0.06369459075581618 |
| 1972 | Lhfpl2 | 0.06346550102845058 |
| 2407 | Icoslg | 0.06341252935966937 |
| 2751 | ENSRNOG00000063679 | 0.06330710980513835 |
| 566 | Pi16 | 0.06316582787254324 |
| 371 | Col4a1 | 0.0631433051938206 |
| 500 | Plaur | 0.06310832652156069 |
| 2872 | ENSRNOG00000069875 | 0.06302663367022257 |
| 695 | Rbp1 | 0.06300949940443334 |
| 990 | Pcolce | 0.06298870773188575 |
| 2733 | Mat2a | 0.0628652513644776 |
| 2033 | Ppic | 0.06276506473566235 |
| 1763 | Mylip | 0.06252611471258653 |
| 1766 | Cercam | 0.06245627037708654 |
| 1961 | Dbn1 | 0.062448632999407325 |
| 475 | Has2 | 0.0622890558600441 |
| 468 | Socs3 | 0.062178071246068035 |
| 2368 | Efemp2 | 0.06202125786180038 |
| 2705 | Vsnl1 | 0.0620021844730019 |
| 1823 | Ckap4 | 0.06199506600959438 |
| 287 | Mt2A | 0.06198857966250003 |
| 877 | Otud1 | 0.06183270609929447 |
| 434 | Cavin2 | 0.061374907709197804 |
| 1357 | Pmepa1 | 0.06135436525155348 |
| 2714 | Slc1a4 | 0.061039324505740455 |
| 295 | Flrt3 | 0.06062415748709653 |
| 1818 | C1qtnf6 | 0.06038554684289397 |
| 2228 | Calml4 | 0.06005416129295045 |
| 956 | Tubb3 | 0.05992840524906179 |
| 2220 | Tent5a | 0.059903352120377285 |
| 290 | Tsc22d1 | 0.05967745570535625 |
| 2438 | Bhlhe41 | 0.05952380438509739 |
| 1542 | Pim3 | 0.05949431984534209 |
| 2535 | Ptch2 | 0.05918054180456389 |
| 1344 | Cotl1 | 0.05917031087039283 |
| 2324 | Ltbp1 | 0.05908843748480655 |
| 1556 | Ier2 | 0.058651298590966044 |
| 2042 | Ebf1 | 0.05858451915185072 |
| 2086 | Wnt11 | 0.05852450765206077 |
| 2523 | Fam114a1 | 0.05849322306962419 |
| 3027 | Adam15 | 0.05845674538942357 |
| 2874 | ENSRNOG00000020119 | 0.0583846570693793 |
| 13 | Pla2g2a | 0.05818598546132246 |
| 1903 | Rnf19b | 0.058148620571088834 |
| 2494 | Apol9a | 0.05794415254790006 |
| 3104 | Vash1 | 0.05779012265869603 |
| 3002 | Bst1 | 0.057721110904743925 |
| 2212 | Copz2 | 0.0576316106511274 |
| 1275 | Ugdh | 0.0575408626479712 |
| 1867 | Angptl2 | 0.05745779950306692 |
| 246 | Gsta1 | 0.057422526419500804 |
| 2115 | Hmgcs2 | 0.057306143794697825 |
| 2656 | Rap2a | 0.056975764790131084 |
| 533 | Ccdc152 | 0.05682372997466344 |
| 331 | Thy1 | 0.05676817215807637 |
| 1091 | C1qtnf5 | 0.05635250016141028 |
| 1586 | Islr | 0.05629504335041707 |
| 2926 | ENSRNOG00000004009 | 0.05625622261421054 |
| 1862 | Pvr | 0.05623296695424952 |
| 1879 | Reck | 0.05622257644154281 |
| 1083 | Gstt3 | 0.05590902686872839 |
| 2406 | Zfp703 | 0.05569868149307901 |
| 2091 | Samd9 | 0.05515440748482729 |
| 2895 | Nfix | 0.055137167476705634 |
| 2992 | Slc4a4 | 0.054962440521437575 |
| 2615 | Mme | 0.054867760485013795 |
| 1784 | Pola2 | 0.05486215068580719 |
| 839 | Angptl4 | 0.05477418664708574 |
| 2112 | ENSRNOG00000032922 | 0.05476904255673515 |
| 2726 | Pdgfd | 0.05475293205951055 |
| 2286 | Fkbp9 | 0.05472658425632622 |
| 1097 | Arid5a | 0.05424536954408138 |
| 390 | Cwc25 | 0.054238855356982855 |
| 1151 | Atf5 | 0.05415631509475754 |
| 693 | Efhd1 | 0.05412703798120476 |
| 1559 | H1f0 | 0.05408155851601783 |
| 2037 | Tgif1 | 0.05407919129973782 |
| 789 | Gpc3 | 0.053852904688950765 |
| 1716 | Pam | 0.05358782665589326 |
| 2256 | Nt5e | 0.05349745391681909 |
| 2196 | Serping1 | 0.053475667114774965 |
| 380 | Ifit3 | 0.05338256429249018 |
| 1710 | ENSRNOG00000065095 | 0.05331864011707893 |
| 2163 | Ap1b1 | 0.05297553752313203 |
| 2791 | Ece1 | 0.0529504409619847 |
| 2219 | C1r | 0.052941592089722565 |
| 1947 | Pcna | 0.052763984280278814 |
| 1002 | Ackr3 | 0.05270809206601909 |
| 3181 | Jade1 | 0.05262292409515377 |
| 2669 | ENSRNOG00000068560 | 0.05248961274532127 |
| 2412 | Fap | 0.052391746522747744 |
| 2340 | Adamts15 | 0.05233795103051903 |
| 3129 | Zfp709l1 | 0.0522924867404716 |
| 1300 | Chst2 | 0.05228229851455712 |
| 793 | Gcsh | 0.05226528086242292 |
| 2896 | Fam126a | 0.05185456832968374 |
| 1956 | Nedd9 | 0.05178200839834994 |
| 1847 | Pxdc1 | 0.05168960772934565 |
| 2387 | ENSRNOG00000054017 | 0.05162160329777758 |
| 2982 | Adcy2 | 0.05156543383831519 |
| 856 | Mfap5 | 0.05154368854839106 |
| 1005 | Col5a3 | 0.051231847002031025 |
| 1891 | Cnst | 0.051097228894237075 |
| 1755 | Abi3bp | 0.05099113000433174 |
| 2946 | Mdfic | 0.05092330704753454 |
| 2124 | Lims2 | 0.050658934464112695 |
| 988 | Hs3st1 | 0.050362852391259115 |
| 163 | Tnfaip6 | 0.050155427549764764 |
| 2380 | ENSRNOG00000064615 | 0.050094931010977825 |
| 1816 | Mrgpre | 0.04996144159974727 |
| 889 | Kcnj8 | 0.04992722055915583 |
| 340 | Mgll | 0.04978163608578455 |
| 1781 | Qprt | 0.04975005200112144 |
| 2069 | Itgbl1 | 0.0495129672133805 |
| 2027 | Ralgds | 0.04947493718368602 |
| 1663 | Tnfaip8 | 0.049400142692192006 |
| 141 | Cxcl9 | 0.04933987538402867 |
| 1152 | Htra3 | 0.049251427102016604 |
| 491 | Rbp4 | 0.049080962964933306 |
| 315 | Col3a1 | 0.049052080185449244 |
| 1067 | Smpd3 | 0.04904273751757458 |
| 577 | Mx1 | 0.048778453635413 |
| 3138 | Thbs3 | 0.04876290863305398 |
| 1761 | Prrx2 | 0.048702509947468026 |
| 433 | Cdkn1c | 0.0485944994551386 |
| 2589 | Maged1 | 0.048332108605909516 |
| 487 | Idi1 | 0.04821905669029594 |
| 2899 | Lvrn | 0.04819822927841995 |
| 3028 | Fads1 | 0.0480587919021446 |
| 1900 | Apcdd1 | 0.04801557372516449 |
| 1279 | PCOLCE2 | 0.04749787302707756 |
| 2905 | Dsel | 0.0474935225472527 |
| 2671 | Bicc1 | 0.04720823984342016 |
| 1514 | Ecm2 | 0.04720777271229966 |
| 2674 | Isca1 | 0.04704055509450537 |
| 2972 | Hspa12b | 0.047012531096732195 |
| 2198 | Dot1l | 0.046998535708378766 |
| 2543 | AABR07044759.1 | 0.04699832346486964 |
| 381 | Pltp | 0.046971249139072434 |
| 1544 | Arl4d | 0.04684677578468052 |
| 2425 | Nectin3 | 0.04682141112112772 |
| 797 | Cebpb | 0.04681892325250417 |
| 2160 | ENSRNOG00000071138 | 0.0467809732877983 |
| 2239 | Lpl | 0.046768953399287036 |
| 1957 | Csn1s2a | 0.046768701450981866 |
| 2724 | Ralgps2 | 0.04667117172969823 |
| 2765 | Kazald1 | 0.04652360582461147 |
| 1774 | C1s | 0.04638244184473671 |
| 1355 | C4b | 0.04617625819240877 |
| 1704 | Cited1 | 0.04573030676350015 |
| 3208 | S100a11 | 0.04563570363072067 |
| 1104 | Gstm6l | 0.04560235121637575 |
| 3182 | Metrnl | 0.04557428489447089 |
| 822 | Cdh13 | 0.0454621721532525 |
| 2728 | Nectin2 | 0.04543804325372295 |
| 3110 | Hoxc8 | 0.045416407154619476 |
| 2491 | Stx11 | 0.04537292766950439 |
| 1567 | Tuba4a | 0.045363402139014596 |
| 1652 | Laptm5 | 0.04535245188228274 |
| 1269 | H1f4 | 0.04485358617632948 |
| 1375 | Lipa | 0.044818561537492495 |
| 2392 | Lgals3bp | 0.0446493387457857 |
| 2088 | Plk3 | 0.04443169179496944 |
| 1207 | ENSRNOG00000064136 | 0.04419508738690903 |
| 1406 | Rhoj | 0.04401623496945248 |
| 1006 | Tgfbi | 0.04393072859711497 |
| 3196 | Fam102b | 0.04386717254362442 |
| 2600 | Baz1a | 0.04385924146030696 |
| 29 | Mgp | 0.04381665704155155 |
| 2062 | Tns3 | 0.04380420279976636 |
| 1690 | Nova1 | 0.04371951959856389 |
| 446 | ENSRNOG00000063824 | 0.04370584332246007 |
| 1835 | AABR07073181.1 | 0.043575538353400285 |
| 1458 | Cd248 | 0.04346425358223291 |
| 2812 | Efhd2 | 0.04312721601127109 |
| 3149 | Zfp622 | 0.043068438928963046 |
| 1945 | Emp1 | 0.0430654385852206 |
| 1496 | ENSRNOG00000059381 | 0.04284492705853097 |
| 2436 | RGD1310507 | 0.04276800995295918 |
| 347 | Nr4a3 | 0.04276500050947679 |
| 1332 | Pcsk6 | 0.042749331982262606 |
| 2588 | Larp6 | 0.04270587212070126 |
| 1568 | Insig1 | 0.04266205556379965 |
| 212 | Hspb1 | 0.042623775288463055 |
| 342 | Phlda1 | 0.04262357711511151 |
| 965 | Olah | 0.04261760055345726 |
| 156 | Ighm | 0.04261351083845951 |
| 1767 | Nfkbie | 0.042608308232377115 |
| 2591 | Ldb2 | 0.042535609668832505 |
| 1801 | Rarres2 | 0.04247314368631637 |
| 544 | Daglb | 0.04244661566149658 |
| 3021 | Me1 | 0.042331134907821144 |
| 2015 | Scd2 | 0.04227834906212985 |
| 698 | Fxyd2 | 0.04223847725199679 |
| 1170 | Nupr1l1 | 0.04223029039455263 |
| 1785 | Pamr1 | 0.04221479756544877 |
| 1122 | Il33 | 0.042017651186642926 |
| 2721 | ENSRNOG00000004137 | 0.041908968262854285 |
| 1864 | Sulf2 | 0.04182335855994277 |
| 1144 | Tspo | 0.041798732124201696 |
| 1297 | Palldl1 | 0.04178636386707761 |
| 1907 | C1qtnf1 | 0.041736169038130065 |
| 2730 | Nab2 | 0.04160363065415499 |
| 358 | Lalba | 0.04146722819559508 |
| 1295 | Mgst3 | 0.04140459981055601 |
| 1954 | ENSRNOG00000015967 | 0.04139098104569543 |
| 2695 | Pla1a | 0.041378056235168395 |
| 1915 | ENSRNOG00000056651 | 0.0413411065547597 |
| 2893 | Phldb2 | 0.04134040197941291 |
| 3157 | Runx1t1 | 0.04131867182463627 |
| 728 | Herc1 | 0.041183420651684635 |
| 3223 | Rtp4 | 0.04114515328406722 |
| 1305 | Cgref1 | 0.04114242680698695 |
| 2545 | Cadm3 | 0.041049072198381414 |
| 2233 | Sdf2l1 | 0.04103894457896004 |
| 39 | Cxcl3 | 0.040902682764495914 |
| 2029 | Snx5 | 0.04090264216721625 |
| 847 | Tf | 0.04085474954618929 |
| 2287 | Higd1a | 0.04085326511212238 |
| 365 | AC128848.1 | 0.0407961324047728 |
| 694 | Rnd3 | 0.040413117773130613 |
| 288 | Dnajb1 | 0.04039946908710043 |
| 1023 | Uap1 | 0.0402127735843017 |
| 1246 | Skil | 0.04011599821343163 |
| 1720 | Glipr2 | 0.04010811613494061 |
| 1844 | Tmem88 | 0.03996757110848892 |
| 1713 | Dusp10 | 0.039887672577018425 |
| 2775 | Usp18 | 0.039787685374535295 |
| 3041 | Tpbg | 0.039629304686725 |
| 1291 | Nrp1 | 0.039599055174693004 |
| 2942 | Mcf2l | 0.039366658053136924 |
| 451 | Plk2 | 0.03933384310467587 |
| 2414 | Ell2 | 0.039151676914209066 |
| 10 | Ccl5 | 0.03908714544921402 |
| 2238 | Lama4 | 0.039085061776838625 |
| 89 | Il1b | 0.03899936476907401 |
| 1515 | Csf2ra | 0.038876084325772005 |
| 1092 | Medag | 0.0388621186763095 |
| 2633 | Cldn4 | 0.03880684563067141 |
| 1983 | Il11ra1 | 0.03836676137199084 |
| 2538 | Nuak1 | 0.03835867712165684 |
| 978 | Socs1 | 0.03834233149155507 |
| 2053 | Csf1 | 0.03818580198141611 |
| 260 | Ltc4s | 0.03812024695763587 |
| 838 | Egr2 | 0.037971979563099126 |
| 1048 | S1pr3 | 0.03780940440955659 |
| 2622 | Tnnt3 | 0.03774341468538117 |
| 152 | Edn1 | 0.037708418113791956 |
| 2635 | Glrb | 0.03754615785555386 |
| 2351 | Adgrd1 | 0.03750656792534442 |
| 1347 | Flna | 0.037371435044503215 |
| 14 | Myoc | 0.037350946371830604 |
| 32 | Hbb-b1 | 0.037329342563072326 |
| 2689 | Fez1 | 0.037309946085481785 |
| 3023 | Nr1d1 | 0.03724143354773045 |
| 166 | Hmgb2l1.1 | 0.03721013823610696 |
| 2900 | Afap1 | 0.0371338490135434 |
| 1966 | Ctsl | 0.03696264420553415 |
| 1105 | Lsp1 | 0.036828319697919415 |
| 2140 | Prkar2b | 0.036799484546287065 |
| 3166 | Slc66a2 | 0.03665344968504346 |
| 828 | Cpxm2 | 0.03661417727486074 |
| 402 | Igfbp5 | 0.03655015426466522 |
| 2161 | Spint2 | 0.03647845519599866 |
| 2763 | Map1a | 0.03645661140548197 |
| 3005 | Cd276 | 0.03633338922842388 |
| 914 | ENSRNOG00000063974 | 0.03606748720941444 |
| 1493 | Map3k20 | 0.03603472376536912 |
| 720 | Fn1 | 0.036004490798428525 |
| 1374 | Flrt2 | 0.03590234467809192 |
| 2771 | Vldlr | 0.03577905526949611 |
| 406 | Folr2 | 0.035707953295652586 |
| 22 | Igfbp3 | 0.03566767821593395 |
| 1298 | ENSRNOG00000066143 | 0.03541527823194899 |
| 2581 | Mab21l1 | 0.035332605181978935 |
| 2114 | Gpm6a | 0.03528453257986976 |
| 945 | Nfkbia | 0.035244111534281225 |
| 2906 | Ltbp3 | 0.03514706545166095 |
| 1577 | Slc25a30 | 0.03512140136111096 |
| 2095 | Nfib | 0.03509779458064129 |
| 1977 | Dmrt2 | 0.03505380114924303 |
| 2251 | Sulf1 | 0.035027762052605355 |
| 2608 | Vnn1 | 0.034981348418406874 |
| 809 | NEWGENE-620180 | 0.03494163120789466 |
| 1923 | Cdc42ep2 | 0.034809260846730694 |
| 1580 | Galnt16 | 0.03478971695203016 |
| 2391 | Cbx4 | 0.03468025285848306 |
| 1202 | Fgf7 | 0.03466005667882551 |
| 2860 | Tsc22d2 | 0.034631343232991635 |
| 1548 | Fbxo30 | 0.03450474331242305 |
| 2413 | Aox1 | 0.03431631311832513 |
| 2661 | Ghr | 0.03420039051185653 |
| 2134 | Plcxd3 | 0.034102397997999676 |
| 2941 | Ptp4a1 | 0.03377141156801319 |
| 645 | Zfand2a | 0.03364287363697584 |
| 321 | Bcl2a1 | 0.03361229767393766 |
| 994 | F3 | 0.033560763590193984 |
| 1892 | Aebp1 | 0.03342433013263291 |
| 481 | Dab2 | 0.03338219393381456 |
| 576 | Ccnd2 | 0.03323492650211851 |
| 1705 | Wnt2 | 0.0331898390851121 |
| 3144 | Fam210b | 0.033043974695423 |
| 521 | Cygb | 0.03289004234281456 |
| 539 | Cd14 | 0.03285913464044138 |
| 1216 | Pcsk5 | 0.03284959803219663 |
| 1776 | Myh9 | 0.03273859619119788 |
| 1611 | Gask1b | 0.03265845725759914 |
| 2079 | Cdo1 | 0.03265068708271583 |
| 2103 | Rilpl2 | 0.03261698819979818 |
| 352 | Col1a1 | 0.03261168143796814 |
| 3125 | Mapk9 | 0.03247398269332637 |
| 2359 | Pbx1 | 0.03245111872674374 |
| 2282 | Slc6a6 | 0.03243891265885286 |
| 2350 | Adora2a | 0.03240116162504528 |
| 46 | Ptx3 | 0.03238777169290014 |
| 2612 | Orai2 | 0.032322845001118206 |
| 1085 | Adcy3 | 0.03227996465830304 |
| 2030 | Trio | 0.03227875124840486 |
| 1702 | Fam107b | 0.03224612506191758 |
| 2898 | Kcnk5 | 0.03224602112614819 |
| 1442 | Mrgprf | 0.03221454086136259 |
| 2542 | Selenom | 0.032197636025217094 |
| 2105 | Irf4 | 0.032155626811098306 |
| 1447 | Slc2a3 | 0.0320476234203394 |
| 2959 | Hmgn2 | 0.03183247420832572 |
| 672 | Col12a1 | 0.03168942293903748 |
| 2090 | Pdpn | 0.031622371927884516 |
| 3085 | ENSRNOG00000029078 | 0.03160575559139668 |
| 773 | Akr1c3 | 0.03158852453566953 |
| 669 | Sectm1b | 0.03152829725695633 |
| 3142 | Nudt9 | 0.03149780447364341 |
| 2839 | Arhgap18 | 0.031491762790173364 |
| 1557 | ENSRNOG00000062707 | 0.03135753792046549 |
| 1182 | Nid2 | 0.0313573016113068 |
| 2687 | Msmo1 | 0.0313270256948086 |
| 2470 | Slc7a8 | 0.03126499715176144 |
| 2005 | Cd44 | 0.0312594959309037 |
| 1452 | Limd2 | 0.031214158890957834 |
| 1533 | Peli1 | 0.031136431055055917 |
| 111 | Igfbp7 | 0.03112693144375948 |
| 466 | Odc1 | 0.031109287750789617 |
| 1197 | ENSRNOG00000064399 | 0.03108498390451987 |
| 2087 | Kdm6b | 0.031034280991896458 |
| 1674 | Scn1b | 0.030953545679588634 |
| 1340 | Osr2 | 0.030904982527486684 |
| 2499 | Znrf2 | 0.030773282351847123 |
| 2255 | Baiap2 | 0.030691989155105293 |
| 2202 | Irs2 | 0.030648881516474855 |
| 2298 | Dgat2 | 0.030629167979589755 |
| 42 | H19 | 0.030588532419247468 |
| 1103 | Tril | 0.03045704068019711 |
| 485 | Tent5c | 0.03025392699215853 |
| 2706 | ENSRNOG00000027520 | 0.03020170036768216 |
| 3112 | Irak3 | 0.030098398292955356 |
| 2732 | Fmnl1 | 0.030075260583394325 |
| 523 | RT1-DMa | 0.02977173055785015 |
| 881 | Bhlhe40 | 0.02976134882881809 |
| 1290 | Igf1 | 0.029757628193803357 |
| 1346 | Snx18 | 0.02951133819374953 |
| 2038 | Angpt4 | 0.0294820687900601 |
| 470 | Sparc | 0.02925259743587442 |
| 1769 | Vps37b | 0.029236147188739722 |
| 1976 | Pak1 | 0.029227647218319476 |
| 205 | Spon2 | 0.02921866879241207 |
| 2518 | Tc2n | 0.02919708713069647 |
| 799 | AABR07002969.1 | 0.029100617092736904 |
| 1399 | Smad7 | 0.029081991988211153 |
| 2958 | Oasl2 | 0.02907776370052227 |
| 1602 | Col6a1 | 0.028995848155790985 |
| 2740 | Plxdc2 | 0.028983253515089666 |
| 2035 | Kdelr3 | 0.02890637828718525 |
| 234 | Hmgb2l1 | 0.028903012470984257 |
| 122 | Rgcc | 0.028886085414222558 |
| 3087 | Fez2 | 0.028884746698734934 |
| 2173 | Rassf9 | 0.028850448789409424 |
| 526 | Gng11 | 0.028819402409470715 |
| 2465 | Arsi | 0.02881119015190918 |
| 1372 | Tifa | 0.02873314796354215 |
| 2360 | Tcn2 | 0.028678950158890597 |
| 49 | Ccl3 | 0.028617041845178506 |
| 450 | Ninj1 | 0.028598082833351093 |
| 3077 | Maged2 | 0.0285830900609157 |
| 1828 | Anpep | 0.028553209944873377 |
| 1639 | Zfhx4 | 0.028506284706021245 |
| 1595 | ENSRNOG00000021403 | 0.028326829582396018 |
| 1093 | Bag3 | 0.02820396084183988 |
| 1039 | Gsn | 0.028172902478990434 |
| 1394 | Serpinb9 | 0.02816528638248665 |
| 648 | ENSRNOG00000065398 | 0.028157014379968925 |
| 1895 | Naf1 | 0.027884036400909917 |
| 2012 | Tcf4 | 0.02779295721864681 |
| 2753 | Rusc1 | 0.02777801103518664 |
| 2650 | Socs2 | 0.02770998722109564 |
| 2701 | Papss2 | 0.02770037734149553 |
| 1107 | H2az1 | 0.02769782011897952 |
| 2441 | Ca8 | 0.02768529379299064 |
| 935 | NEWGENE-1308171 | 0.027446240961141552 |
| 1960 | Ackr2 | 0.027397553294217523 |
| 1292 | Timp1 | 0.027369624599073453 |
| 1469 | Dpp4 | 0.02734921571462002 |
| 2813 | Anp32e | 0.02734548906595117 |
| 2376 | Resf1 | 0.02733209566242746 |
| 3059 | Fzd4 | 0.02728756213077682 |
| 2528 | Nol3 | 0.027265769624260097 |
| 776 | Map1b | 0.027208196651288326 |
| 333 | Cthrc1 | 0.027146659102819597 |
| 2439 | Fkbp10 | 0.02691325745710609 |
| 2749 | Pde7b | 0.026827382315644556 |
| 575 | Ecrg4 | 0.026718775965744818 |
| 3176 | Galr2 | 0.026593352208048926 |
| 2293 | Lsamp | 0.026463062553152644 |
| 1419 | Mmp27 | 0.02644148647422484 |
| 2936 | Tfip11 | 0.026360630477855834 |
| 1167 | Fam43a | 0.02633071025867045 |
| 2780 | Cpq | 0.026298096547271513 |
| 2078 | Igtp | 0.026285712564127844 |
| 2337 | Bcl2l11 | 0.026275802357571952 |
| 768 | Oasl | 0.026182558727507757 |
| 357 | Rarres1 | 0.026182499450382424 |
| 1965 | Gstp1 | 0.02609581749130177 |
| 3175 | Sema3a | 0.02605573796491282 |
| 2720 | Cmahp | 0.026043397762101173 |
| 550 | Thrsp | 0.02595939440956105 |
| 2854 | Cyba | 0.025957964632998914 |
| 323 | Cyb561a3 | 0.02587337043613032 |
| 1276 | RGD1306556 | 0.0258316918264877 |
| 2843 | Slc9a3r1 | 0.025830136561478506 |
| 2335 | Fbxo32 | 0.025804621070279074 |
| 110 | Rgs1 | 0.025760812849981087 |
| 1875 | Map3k8 | 0.025744540235636326 |
| 3040 | ENSRNOG00000064568 | 0.02569402945585176 |
| 2640 | Tubb4b | 0.025598352956494076 |
| 3127 | Serpinb6a | 0.025594857763285024 |
| 2814 | Ipmk | 0.025495425940813526 |
| 753 | Cp | 0.02539811461980954 |
| 427 | Gadd45b | 0.025374094698325453 |
| 2524 | Cpne8 | 0.02529497974604258 |
| 2886 | Paqr6 | 0.02514336810105361 |
| 845 | Zc3h12a | 0.025029609712708505 |
| 2013 | Siah2 | 0.02502223121000207 |
| 508 | Col1a2 | 0.024875850257677662 |
| 633 | Ctsb | 0.024857764177888278 |
| 231 | RT1-Da | 0.024826413383778414 |
| 3204 | Shc2 | 0.024616013948795562 |
| 459 | Ttll7 | 0.024518362156500433 |
| 2326 | Afdn | 0.024272449982920333 |
| 2448 | Rab30 | 0.024254307126866548 |
| 2133 | Runx1 | 0.024231942099970413 |
| 1270 | Fgf18 | 0.024115907560764052 |
| 2315 | Adamtsl4 | 0.0240659299038219 |
| 1035 | Fmo1 | 0.02405648815550859 |
| 927 | Boc | 0.02395946224731289 |
| 210 | Penk | 0.02392634597722713 |
| 3095 | ENSRNOG00000068096 | 0.023918946314522346 |
| 1858 | Cybrd1 | 0.023883727858985786 |
| 3169 | Rora | 0.023883289332209456 |
| 1338 | Nid1 | 0.02381024499258896 |
| 3111 | Sytl2 | 0.023805228385892427 |
| 564 | Myc | 0.023767515171980752 |
| 2216 | Gas7 | 0.023677651824079108 |
| 1498 | Slc5a3 | 0.023586363327250476 |
| 2009 | Resp18 | 0.02354800273844327 |
| 3060 | Hyal2 | 0.0234860941577335 |
| 2925 | Bdkrb2 | 0.02347223732050556 |
| 2116 | Rxfp1 | 0.023380855461048927 |
| 930 | Ccdc80 | 0.02331245855431477 |
| 2479 | Iqcn | 0.023308693585734834 |
| 3014 | Rabgef1 | 0.02320775182583701 |
| 3123 | Acot7 | 0.023188620391974567 |
| 2986 | Tmem45al | 0.02284727709509527 |
| 1584 | Ucp2 | 0.02280285598373003 |
| 389 | Hbegf | 0.0227052502517556 |
| 2369 | Nrip1 | 0.022692907555984722 |
| 1686 | Hsd11b2 | 0.0226826545499992 |
| 2458 | Tgfb1 | 0.022647571775273496 |
| 1263 | Col6a3 | 0.022521455830006032 |
| 1485 | ENSRNOG00000066846 | 0.02240444949907511 |
| 498 | ENSRNOG00000030021 | 0.02234378259014015 |
| 2131 | Fbln5 | 0.022154298918668602 |
| 2627 | Hvcn1 | 0.022152189837487495 |
| 1459 | Aldh3a1 | 0.02213022811854961 |
| 2023 | Rin3 | 0.02211957341287791 |
| 2506 | Osmr | 0.022084088342528785 |
| 868 | Tfpi | 0.02208244119150612 |
| 715 | Ly86 | 0.021889056426160985 |
| 2605 | Sptbn1 | 0.021793191069992283 |
| 2183 | Rasal2 | 0.021725135295975893 |
| 2799 | ENSRNOG00000066365 | 0.021719618714281082 |
| 1513 | Sct | 0.021647765000428403 |
| 1212 | Mycl | 0.021645850944982784 |
| 247 | RT1-Ba | 0.02162290482918304 |
| 2361 | Kctd12 | 0.021615610177947372 |
| 1935 | Pld3 | 0.021499403965842677 |
| 1323 | Il4r | 0.02146270557508065 |
| 1814 | Adamts4 | 0.021365649304450604 |
| 803 | Pla2g2d | 0.021240751170936475 |
| 755 | Gfpt2 | 0.02115722020413424 |
| 1410 | Bmp4 | 0.02107371862806084 |
| 3172 | Oaf | 0.021019918540912848 |
| 1800 | Slc1a5 | 0.020958721028517015 |
| 1405 | Hsp90aa1 | 0.020933086733328093 |
| 882 | Hsd11b1 | 0.020916651422151534 |
| 278 | ENSRNOG00000003984 | 0.02079232982573232 |
| 2859 | Cideb | 0.020687323139901678 |
| 440 | MGC105649 | 0.02064785680824168 |
| 2432 | Lig1 | 0.020633263829934856 |
| 3122 | Zfp36l1 | 0.020608901005804953 |
| 72 | RGD1565462.1 | 0.020559000675554065 |
| 1658 | Pdgfrl | 0.020536053061155524 |
| 3101 | Ptgfrn | 0.020520208791565405 |
| 2620 | Kcnab1 | 0.020451355229936387 |
| 2561 | Tgfb3 | 0.02037749640630987 |
| 1013 | Hist1h2bq | 0.02037354436032182 |
| 2107 | Pycard | 0.020329703420110302 |
| 2917 | Sema4c | 0.020323445251065032 |
| 301 | Bgn | 0.02016782183395463 |
| 452 | Eng | 0.020157887374810474 |
| 2513 | Stard10 | 0.02008756766071869 |
| 573 | Fcgr2a | 0.02006722137097884 |
| 899 | Pparg | 0.02004644347871257 |
| 1815 | Hspa2 | 0.020000884259924114 |
| 1599 | Slc25a25 | 0.019939998932743338 |
| 1471 | Hpse | 0.01993324729986344 |
| 2831 | Hmx1 | 0.01992974126162597 |
| 1887 | Pth1r | 0.019887869595752797 |
| 2106 | Pde4c | 0.019821971093749884 |
| 2096 | Sspn | 0.019581109053370606 |
| 911 | Ifi30 | 0.019580178407605717 |
| 2181 | Setdb2 | 0.01949763894936623 |
| 1166 | Osr1 | 0.0194875252938467 |
| 1147 | Adgre5 | 0.01942706336665559 |
| 2639 | Fabp3 | 0.01941715641086114 |
| 949 | Ehd4 | 0.019415481004613305 |
| 2467 | Lrrfip1 | 0.019389571651018112 |
| 2440 | Pcdh18 | 0.01931455433356371 |
| 2988 | Zrsr1 | 0.01929695055125033 |
| 2989 | Lpar4 | 0.019249703242832913 |
| 1741 | Fosl1 | 0.01909180969903619 |
| 2624 | Prelp | 0.01907968982070741 |
| 37 | Sncg | 0.019048249854466036 |
| 622 | Icam1 | 0.019003972245033342 |
| 1607 | AABR07044388.2 | 0.01899601251300485 |
| 83 | Rnase1 | 0.01898089173830367 |
| 2292 | Fndc5 | 0.018974084056832697 |
| 1385 | Htra1 | 0.01894834629657053 |
| 1038 | Tmem176a | 0.018938380690799943 |
| 723 | Kcne4 | 0.018874967830010685 |
| 2938 | Ephb4 | 0.018865201093857815 |
| 629 | ENSRNOG00000070904 | 0.018735194248598 |
| 2708 | Ifi47 | 0.018677305984677706 |
| 639 | Thbd | 0.01863388408438419 |
| 1268 | Fbln2 | 0.018621247846193182 |
| 2970 | Abca9 | 0.018616661360778775 |
| 2969 | Marcks | 0.018589400695683534 |
| 2135 | Ptpn13 | 0.01858294355611554 |
| 1317 | Sema3c | 0.018566860806602026 |
| 730 | Cldn3 | 0.018461347463057706 |
| 492 | Rflnb | 0.018423470486568456 |
| 821 | Pdk4 | 0.018373198132968324 |
| 2304 | Mamdc2 | 0.018336949085941495 |
| 1981 | Chl1 | 0.01831078025777979 |
| 341 | F13a1 | 0.01823061546820552 |
| 1365 | Smc4 | 0.01821375087461221 |
| 2354 | Chrdl1 | 0.018202328129609806 |
| 801 | Frmd6 | 0.018165496853759754 |
| 126 | Cd24 | 0.0181551737336113 |
| 998 | Arrdc3 | 0.018117954416317902 |
| 2716 | Pdgfc | 0.018098431489947102 |
| 2389 | Tgfb1i1 | 0.018032732274945732 |
| 149 | Ccl7 | 0.0179900811702969 |
| 2208 | Slc12a2 | 0.017933526389500014 |
| 2024 | Rab7b | 0.017906320598313943 |
| 172 | Rnase2 | 0.017881545815292204 |
| 664 | Cald1 | 0.01783384110794344 |
| 519 | Col8a1 | 0.017787523878560765 |
| 1694 | Hpgd | 0.017735428502999057 |
| 626 | Prdx5 | 0.01764822967666916 |
| 3193 | Tspan9 | 0.017645108320969407 |
| 2517 | Dusp22 | 0.01760512570614813 |
| 93 | Cpe | 0.017602431854423298 |
| 386 | Ackr1 | 0.01756621616577652 |
| 1168 | Ddit3 | 0.017545144240289808 |
| 2475 | Klf10 | 0.017473112070588794 |
| 1281 | Cavin3 | 0.017451811104133297 |
| 2249 | Nfe2l1 | 0.017440681753325273 |
| 1893 | Dnajb4 | 0.017346452486196123 |
| 38 | Hba-a2 | 0.017326090001956413 |
| 3173 | Cks1b | 0.017320272668359865 |
| 2221 | Phyhd1 | 0.017285118815388252 |
| 1906 | S100a16 | 0.017268611412636303 |
| 2521 | Igf2r | 0.01724978049559049 |
| 2693 | Slc1a3 | 0.01724033834961291 |
| 940 | Efcc1 | 0.017190047416061408 |
| 2932 | Diaph3 | 0.017176884435522705 |
| 3227 | ENSRNOG00000062374 | 0.017145219605402322 |
| 1765 | Bace2 | 0.017095752149379417 |
| 1456 | Tmem100 | 0.017081742475601232 |
| 943 | ENSRNOG00000023151 | 0.017064967329385346 |
| 2126 | Noct | 0.017041223010585152 |
| 462 | Epcam | 0.01700425618042988 |
| 3186 | Sgms1 | 0.016988037201938304 |
| 2394 | Hivep3 | 0.016939820868670075 |
| 2264 | Atp2b4 | 0.01692321223073334 |
| 2325 | Egln3 | 0.016919057534793114 |
| 366 | Timp3 | 0.01690560936195086 |
| 58 | Tm4sf1 | 0.016848264695388755 |
| 51 | Tpm2 | 0.01679493186046563 |
| 901 | Gas6 | 0.016794627234068625 |
| 1708 | Golm1 | 0.01678363884547661 |
| 1378 | Bok | 0.01675643844542052 |
| 2108 | RGD1304884 | 0.016754793809480015 |
| 918 | ENSRNOG00000062927 | 0.016704881372082245 |
| 2144 | Fkbp11 | 0.016696714288675085 |
| 399 | Mafb | 0.016489297559425846 |
| 1495 | H1f2 | 0.016471859673282525 |
| 1094 | Fhl2 | 0.0164248398184859 |
| 31 | Crabp1 | 0.016402990779700744 |
| 3061 | Vegfc | 0.01639907013226083 |
| 763 | Cd200 | 0.01638052041142762 |
| 2861 | Rab33a | 0.016308757153969098 |
| 173 | Cyp26b1 | 0.01628981392717535 |
| 2001 | MGC108823 | 0.016276316922925325 |
| 567 | Cdc20 | 0.016250656414424546 |
| 1558 | Osgin1 | 0.01618545351699346 |
| 657 | Id2 | 0.01613442879988073 |
| 318 | Gadd45g | 0.01607606644757032 |
| 1898 | ENSRNOG00000067560 | 0.016047819142700794 |
| 2070 | Slc43a2 | 0.016023330701899596 |
| 2002 | Gng2 | 0.01601845376502131 |
| 3200 | Ldb3 | 0.015991662077427388 |
| 1970 | Mxra8 | 0.01593976065154212 |
| 1643 | Smc2 | 0.015939130351349952 |
| 756 | Crip2 | 0.015917502684881683 |
| 1868 | Itpkc | 0.015890617706039323 |
| 2853 | Mx2 | 0.015872870124299908 |
| 1634 | Rtn1 | 0.01585585230607666 |
| 2254 | Arhgap24 | 0.015836600284139125 |
| 1508 | Vasn | 0.015670215023866577 |
| 3170 | Litaf | 0.015504010480812663 |
| 1434 | Loxl2 | 0.01541945726200623 |
| 2990 | Prkcd | 0.015281859623603015 |
| 2662 | Fnbp1l | 0.015164420272654851 |
| 336 | Mal | 0.015106051889263736 |
| 555 | Mdk | 0.01507366843177102 |
| 165 | Thbs1 | 0.015017924133421642 |
| 1134 | ENSRNOG00000003809 | 0.01501596219559248 |
| 2137 | Plcb1 | 0.014883466440951176 |
| 2225 | Tspan2 | 0.014843925687521364 |
| 2127 | Slc2a1 | 0.014779064284109987 |
| 2628 | Adam19 | 0.014754691918170495 |
| 1004 | Arid5b | 0.01472637641172822 |
| 2786 | Acox1 | 0.014667112528815581 |
| 305 | Selenop | 0.014634368364054257 |
| 2754 | Csgalnact1 | 0.014562321288625078 |
| 849 | Gch1 | 0.014554535979720989 |
| 3214 | Enpp1 | 0.014550579456299638 |
| 69 | Cxcl10 | 0.014466397387360434 |
| 1329 | Ets2 | 0.01440739394979397 |
| 2205 | ENSRNOG00000067579 | 0.014376183756566189 |
| 826 | Fst | 0.014364244739288237 |
| 3212 | Rnf122 | 0.014361333027977166 |
| 1248 | Dusp6 | 0.014349785624429548 |
| 1489 | Col5a2 | 0.014310267771759571 |
| 3049 | Mbd1 | 0.014290296498852188 |
| 2379 | Adamts2 | 0.014282664291637737 |
| 1989 | Axl | 0.014261383629321148 |
| 1254 | Srgn | 0.014237997739178223 |
| 1794 | Hilpda | 0.014219711316898084 |
| 2478 | Tyms | 0.014196262836608674 |
| 736 | Spock2 | 0.014149499769897364 |
| 507 | Mgst2 | 0.014096078850227678 |
| 261 | ENSRNOG00000067254 | 0.014086581513127187 |
| 2453 | Actb | 0.014075759530249365 |
| 2623 | Msrb3 | 0.014063033491404402 |
| 504 | Gprc5a | 0.014021599217480715 |
| 532 | Tcim | 0.01401101091264318 |
| 796 | Synm | 0.01400429506794993 |
| 2277 | Ptpn1 | 0.013977761978165248 |
| 887 | Dio2 | 0.013834311776415711 |
| 3082 | Pfkfb3 | 0.013833778488375732 |
| 3037 | Sdad1 | 0.01376566510263237 |
| 30 | Jchain | 0.013742234117195743 |
| 2677 | Cep295nl | 0.013705182667526024 |
| 2884 | Gss | 0.013653808967686299 |
| 2829 | Lgals1 | 0.013571831670242764 |
| 905 | Snai1 | 0.013538877143179816 |
| 875 | Tnfsf9 | 0.013532811488419744 |
| 1700 | Dock9 | 0.013519226133462688 |
| 2887 | Olfml1 | 0.013513310399969163 |
| 766 | Aif1 | 0.013511444259520534 |
| 1552 | Crip1 | 0.013491847824804601 |
| 1235 | Olfm1 | 0.013456019853271625 |
| 1222 | Grem2 | 0.013427208565968293 |
| 1692 | Pdia5 | 0.013408835342640212 |
| 1131 | Dpt | 0.013396533322660607 |
| 2795 | ENSRNOG00000064025 | 0.013361160432466244 |
| 2734 | Msc | 0.013196125932094311 |
| 1822 | Twist1 | 0.013128991338098651 |
| 890 | Col4a2 | 0.013113719464265611 |
| 2486 | Psip1 | 0.013105310378830677 |
| 2271 | Septin11 | 0.013075550784095127 |
| 1271 | Fndc1 | 0.013056831760638269 |
| 437 | Mrc1 | 0.01299732519395927 |
| 2655 | Dctpp1 | 0.012964309481262478 |
| 1337 | Lif | 0.012926096322451905 |
| 1201 | Tlr2 | 0.012907912823185642 |
| 2065 | Rgma | 0.012901346821159702 |
| 1204 | Rxfp3 | 0.012898220160592909 |
| 2643 | Pid1 | 0.012869024896613814 |
| 3088 | Prim1 | 0.012840987578116995 |
| 2186 | Col5a1 | 0.012823342546232946 |
| 785 | Lrrc15 | 0.01279549530182893 |
| 2867 | Rasa3 | 0.012771304321989389 |
| 953 | Fcer1g | 0.012768408570412157 |
| 3238 | Hoxd9 | 0.012768092249244047 |
| 3164 | Lonrf3 | 0.012726154948346379 |
| 1803 | Chd7 | 0.012724409268096908 |
| 1650 | Ciart | 0.012706301818105397 |
| 3078 | Col6a6 | 0.012670940094456323 |
| 898 | Gpr183 | 0.012664996255882022 |
| 1020 | Ccdc168 | 0.012603965318616253 |
| 3234 | Rspo3 | 0.012582806688277652 |
| 2399 | Arhgap6 | 0.012561803178435003 |
| 2051 | Klf4 | 0.012474079083345392 |
| 886 | Vcan | 0.012470429966574489 |
| 1284 | Igsf10 | 0.01246318706734236 |
| 2067 | Sdc2 | 0.012446038503258112 |
| 1225 | Stom | 0.012410938016604872 |
| 1116 | Ly49si2 | 0.012397330389411059 |
| 2026 | Prss23 | 0.012365284415624005 |
| 1255 | Traf1 | 0.012338519459862429 |
| 3126 | Abr | 0.012319303270618646 |
| 2908 | ENSRNOG00000063760 | 0.012317068789440874 |
| 71 | Mustn1 | 0.012306417422787371 |
| 1228 | Serpinh1 | 0.012292018790640716 |
| 2191 | ENSRNOG00000070985 | 0.01222596759561105 |
| 3007 | Svil | 0.012216584223077778 |
| 2143 | Dnm3 | 0.012191837390884721 |
| 3216 | Prpf40b | 0.012167304905399863 |
| 1150 | AABR07054614.1 | 0.012157418899428474 |
| 1629 | Colec12 | 0.012116528458761024 |
| 2707 | Tnfrsf21 | 0.012097445879525557 |
| 1645 | Fbxo5 | 0.01204877268610231 |
| 227 | Slfn4 | 0.012028811643611765 |
| 1905 | H2ax | 0.012028411137698381 |
| 2725 | Kbtbd11 | 0.012004556669320772 |
| 2616 | Hacd4 | 0.011995461170728559 |
| 2496 | Grb10 | 0.011988513845076017 |
| 2207 | Dkk2 | 0.01197438748050122 |
| 2629 | Matn4 | 0.011932254669602953 |
| 1433 | Lcp1 | 0.011922777435838937 |
| 2852 | ENSRNOG00000067693 | 0.011911323889134089 |
| 2901 | Rfc2 | 0.011893107606727044 |
| 1596 | Mthfd2 | 0.01185025312147513 |
| 80 | Ccl11 | 0.011830474542683357 |
| 955 | Plek | 0.01183043733178201 |
| 790 | Adgre1 | 0.011830341024529706 |
| 2510 | Qpct | 0.011776764805999578 |
| 3139 | Isg20 | 0.011771986781685471 |
| 742 | Tiparp | 0.011764136662607956 |
| 2904 | Slc16a1 | 0.011732023430431327 |
| 974 | ENSRNOG00000070737 | 0.011729435079452151 |
| 2401 | Snai2 | 0.011721588877592747 |
| 6 | Cpa3 | 0.01172065831583921 |
| 3162 | Slc16a11 | 0.011689655018385734 |
| 2525 | Myo5a | 0.01166460612493167 |
| 3235 | Gulp1 | 0.011654940021234295 |
| 1435 | Spi1 | 0.01161810068751159 |
| 1176 | Ezh2 | 0.011609073873765103 |
| 1171 | Ptprc | 0.011529660814325968 |
| 175 | Chi3l1 | 0.011518105463593078 |
| 1787 | Mmp2 | 0.011476660365293708 |
| 1597 | Calhm6 | 0.011446031226773658 |
| 1660 | Sema4a | 0.011439479160336136 |
| 1721 | Emx2 | 0.01139695291020066 |
| 820 | Il34 | 0.011393159010052275 |
| 565 | ENSRNOG00000064142 | 0.011337159495449958 |
| 2230 | Dab2ip | 0.01132628679306704 |
| 1499 | Gpm6b | 0.0112906394703834 |
| 650 | Coro1a | 0.011266687599767716 |
| 1554 | Milr1 | 0.011254452890864764 |
| 589 | Krt18 | 0.011244431612117666 |
| 1638 | Pdgfra | 0.011225238489082127 |
| 603 | Ets1 | 0.011215295311344601 |
| 2165 | ENSRNOG00000003929 | 0.011128489709211094 |
| 1380 | Plac9 | 0.011123400496746875 |
| 1036 | Sema5a | 0.011099646432382388 |
| 738 | Ltbp4 | 0.011098107170536976 |
| 2246 | Cdkl1 | 0.011094526460716847 |
| 132 | Il10 | 0.01108081553772066 |
| 3017 | Soat1 | 0.010995700409390982 |
| 1855 | Lyn | 0.010972646117707341 |
| 3038 | Rnpep | 0.010933581365432841 |
| 3042 | Rfc3 | 0.010931486968805394 |
| 2849 | Tbx18 | 0.010925338526880298 |
| 1314 | ENSRNOG00000063437 | 0.010922770257130388 |
| 707 | Zdbf2 | 0.010896634999207207 |
| 57 | AC112568.1 | 0.010846811052276754 |
| 1047 | Aopep | 0.010824417060316935 |
| 1917 | Prex2 | 0.010801696164517885 |
| 2913 | Flnb | 0.010763420044891376 |
| 2584 | Ntn1 | 0.010763146368656567 |
| 1687 | Nrep | 0.010745019478959594 |
| 913 | Cd68 | 0.010722090388943898 |
| 1455 | Syngr2 | 0.010700985551981247 |
| 1183 | Mxd1 | 0.010697331607646809 |
| 27 | Ccl17 | 0.010693150804034383 |
| 2393 | Fam110a | 0.010676658188969387 |
| 2153 | Gpsm3 | 0.010670388080117721 |
| 2498 | Twf2 | 0.010663874654352824 |
| 2897 | Hmga1 | 0.010658198484084084 |
| 800 | Ctsc | 0.010653985799614064 |
| 2339 | AABR07007032.1 | 0.010652565033773329 |
| 602 | Cfp | 0.010608618136694059 |
| 2025 | Ubc | 0.010561788545636781 |
| 317 | Rgs2 | 0.010548491354035138 |
| 2652 | Cdc14a | 0.01052162302272094 |
| 966 | Vit | 0.010447305937215073 |
| 1712 | Ifitm3 | 0.010435337875882956 |
| 1444 | Lilrb3a | 0.01043015260506909 |
| 2349 | Frmd4b | 0.010428914030242799 |
| 583 | Krt15 | 0.010417038028245501 |
| 143 | Foxq1.1 | 0.01041078404338053 |
| 692 | Atp1b1 | 0.010393596969590913 |
| 480 | Ddit4 | 0.01037047067926316 |
| 2312 | Zfp503 | 0.010366176380643702 |
| 1231 | Aard | 0.010300983794876147 |
| 510 | Gja1 | 0.010262041228243495 |
| 1723 | Tcf19 | 0.01023316443578614 |
| 1069 | Knl1 | 0.01021868708306488 |
| 2880 | Cep290 | 0.010187673996129243 |
| 1683 | Crim1 | 0.010186727802313908 |
| 1969 | ENSRNOG00000059827 | 0.010183894038962952 |
| 1736 | Tmem47 | 0.010096311443688409 |
| 1145 | Il18 | 0.010078539886230904 |
| 1625 | ENSRNOG00000003228 | 0.010077697244933858 |
| 2493 | Dmd | 0.01007658843585986 |
| 1430 | Msrb1 | 0.010025993443586692 |
| 2102 | Sema3d | 0.010022343446188381 |
| 1045 | ENSRNOG00000065273 | 0.009766666603694522 |
| 2710 | Etv5 | 0.00973968753733204 |
| 1063 | Fstl1 | 0.009684713118127651 |
| 2821 | Slc20a1 | 0.00967386527813607 |
| 102 | Serpine1 | 0.009653424673076071 |
| 1096 | Samhd1 | 0.009641129806108449 |
| 1501 | ENSRNOG00000047931 | 0.009594148344824626 |
| 426 | Crabp2 | 0.009582029504097535 |
| 2370 | Ssc5d | 0.00957235708941831 |
| 3143 | Rap1gap2 | 0.009546044576461132 |
| 420 | Ms4a4a | 0.009492496077595444 |
| 1214 | ENSRNOG00000063787 | 0.009368603526370938 |
| 2021 | Myo1g | 0.009337405100908596 |
| 2468 | ENSRNOG00000067673 | 0.009321643998049961 |
| 798 | Tcf7 | 0.009321622319055242 |
| 3024 | Tnfrsf1b | 0.009283842125304012 |
| 2870 | NEWGENE-6497122 | 0.00928110746607348 |
| 255 | Rasl11a | 0.009280395139901493 |
| 2492 | Nxn | 0.0092075286140396 |
| 2150 | Shroom3 | 0.009194132435648386 |
| 2121 | Nuak2 | 0.00918413734975577 |
| 1294 | Ncf4 | 0.009183526060728722 |
| 2828 | Cited4 | 0.00917528991909133 |
| 1264 | ENSRNOG00000011478 | 0.009164924099088957 |
| 1071 | Fxyd5 | 0.009135329696893839 |
| 2083 | Arhgdib | 0.009119451998565401 |
| 1927 | Chst1 | 0.009115662515136768 |
| 2076 | Gsc | 0.009112360349825807 |
| 26 | Fabp4 | 0.009112072740537887 |
| 372 | Trem2 | 0.009107007796782422 |
| 252 | Nr4a2 | 0.009091663631534467 |
| 160 | Osm | 0.009076240548048252 |
| 1052 | Vcl | 0.009048895352441622 |
| 2476 | Fads3 | 0.00903076084018759 |
| 493 | Serpinb6b | 0.009030627618394332 |
| 3066 | Rhou | 0.009008256801525918 |
| 1056 | Mt1 | 0.008996482152425947 |
| 2827 | Ninj2 | 0.008968831446925594 |
| 2258 | Adcy6 | 0.008966157247084768 |
| 2590 | Gnpda1 | 0.008945779006071772 |
| 2182 | Diras3 | 0.008939296267744985 |
| 1988 | Olfml2b | 0.008918997958533919 |
| 2110 | P2ry14 | 0.008912755317681289 |
| 2018 | Sod3 | 0.008894656535839757 |
| 36 | Apod | 0.008892271673738922 |
| 2138 | Efna1 | 0.008885830818503984 |
| 370 | Lyve1 | 0.008874913643667359 |
| 2679 | Rprml | 0.008857218796740101 |
| 1393 | ENSRNOG00000069188 | 0.008807563773043127 |
| 1137 | Peg3 | 0.008804295559551725 |
| 1536 | Nrros | 0.008675079350121481 |
| 975 | H4f3 | 0.008656844251288633 |
| 1866 | Vstm4 | 0.008614979197836336 |
| 245 | Ltb | 0.008584927767935185 |
| 3198 | Stbd1 | 0.008554626333786019 |
| 2802 | Mns1 | 0.008543297873420477 |
| 2596 | Ppp1r13b | 0.008539280214800916 |
| 1115 | Meox2 | 0.008529967564668207 |
| 403 | Ccr2 | 0.008498816030082972 |
| 2050 | Tm6sf1 | 0.008497761914889317 |
| 1971 | Irf5 | 0.008494985267055707 |
| 2260 | Irx3 | 0.008478263710722193 |
| 2411 | Dact1 | 0.008440908967499185 |
| 2449 | Scara5 | 0.008439507074278315 |
| 1224 | Mef2c | 0.008427419912089642 |
| 2395 | Lpxn | 0.00839101421342317 |
| 819 | Mtss1 | 0.008377673701087305 |
| 1068 | Arl5b | 0.00832571215999534 |
| 2991 | AC131360.1 | 0.008284591550307167 |
| 1804 | Acer2 | 0.008279277011319931 |
| 1565 | Oaz2 | 0.008275575891786402 |
| 621 | Alcam | 0.0082472179868067 |
| 1764 | Atad2 | 0.008241547382885586 |
| 1795 | Ckap2 | 0.008240971688609586 |
| 2022 | Amotl2 | 0.008227930886531476 |
| 1732 | Cdkn2c | 0.008194214179179139 |
| 2398 | Rgs10 | 0.008189461514603555 |
| 1504 | ENSRNOG00000066282 | 0.00818479884096476 |
| 2068 | Wtip | 0.008137332207579786 |
| 2663 | Lmnb1 | 0.008132771331581096 |
| 1780 | Col6a2 | 0.008126745245684883 |
| 1505 | Pard6g | 0.008100959015344918 |
| 2715 | Tmem38b | 0.008095003731155062 |
| 631 | Trib1 | 0.008091303398515713 |
| 2274 | ENSRNOG00000014851 | 0.008069768045766666 |
| 3010 | Dipk1b | 0.008046668986710324 |
| 547 | Rnd1 | 0.00801734239742563 |
| 219 | Cxcr4 | 0.007985540530641756 |
| 2280 | Tbxas1 | 0.007978524165398227 |
| 1108 | Bambi | 0.007975358883583913 |
| 2533 | Prnp | 0.007957560288571681 |
| 1353 | Ahr | 0.007945118663763623 |
| 1302 | ENSRNOG00000045992 | 0.007941348552258013 |
| 2516 | ENSRNOG00000070062 | 0.007893350686775744 |
| 2426 | Nckap1l | 0.007844521348144819 |
| 2944 | Oas1k | 0.007830109577415851 |
| 2700 | Hsd17b1 | 0.007804082705142205 |
| 70 | Esm1 | 0.007767644627359883 |
| 206 | Ptp4a3 | 0.007744738811996062 |
| 2452 | P2ry2 | 0.007732743763615107 |
| 2410 | ENSRNOG00000029191 | 0.0077135920592156584 |
| 2527 | Ptpre | 0.007687278268085497 |
| 2288 | Clca1 | 0.007685445369887321 |
| 2855 | Inpp5d | 0.007675639493624861 |
| 2536 | Col16a1 | 0.007649063528346052 |
| 469 | Gjc1 | 0.007643033466270079 |
| 1470 | Unc93b1 | 0.007629175070137669 |
| 712 | Giot1 | 0.007628102912453152 |
| 1129 | Tp53rk | 0.007591685776891618 |
| 931 | Glrx | 0.007525048415394149 |
| 2851 | Gltpd2 | 0.007521215318444437 |
| 2976 | Hmgcr | 0.007516778632008727 |
| 65 | Gdf15 | 0.007495917053360822 |
| 580 | Neurl3 | 0.007490099688845558 |
| 3099 | Notch4 | 0.007472256411978391 |
| 1031 | Cdca5 | 0.007464406196922426 |
| 926 | Dusp5 | 0.007459865383929695 |
| 2338 | Fam241a | 0.007448742568940752 |
| 2222 | ENSRNOG00000062871 | 0.007369751473561285 |
| 542 | Il6st | 0.007356511515199945 |
| 1102 | Cyp7b1 | 0.007340823544149283 |
| 902 | Tagap | 0.00726070624686634 |
| 1073 | Avpr1a | 0.0072534146769415815 |
| 1114 | AABR07072096.1 | 0.007181790857120307 |
| 612 | Pdgfrb | 0.0071554238250830515 |
| 1612 | AABR07008309.1 | 0.0071166545584192276 |
| 429 | Hopx | 0.007115328245142846 |
| 269 | Clec14a | 0.007111572230498229 |
| 128 | Slpil3 | 0.007102594207174406 |
| 2471 | Plscr2.1 | 0.00710222971151561 |
| 2209 | Gpc1 | 0.007061652878450899 |
| 2610 | Syne2 | 0.007040518577159415 |
| 2544 | Snn | 0.007039114475521423 |
| 2447 | Gpat3 | 0.007013495762809116 |
| 3093 | Pitx2 | 0.007005575686492535 |
| 2097 | Sema6d | 0.0069864995118064065 |
| 2842 | Hmgn3 | 0.006935723551070017 |
| 3091 | Rrm1 | 0.006920024403071171 |
| 2 | Mcpt1l1 | 0.0069176608602845155 |
| 686 | Ms4a7 | 0.006905337099031761 |
| 1367 | Basp1 | 0.006858370122869002 |
| 1749 | Jcad | 0.006804970937411541 |
| 1510 | Pou3f1 | 0.0067738755702322125 |
| 3167 | Kif15 | 0.006748518935255687 |
| 578 | Cystm1 | 0.006748038698469451 |
| 300 | Lcn2 | 0.006731824132384285 |
| 2819 | Aox4 | 0.006729165232382687 |
| 62 | Thbs4 | 0.0066546190280387055 |
| 214 | Cd83 | 0.006646868649230335 |
| 136 | Areg | 0.006640724028290598 |
| 258 | Pecam1 | 0.006632287459374624 |
| 2267 | Lama2 | 0.006625116619706654 |
| 781 | Crispld2 | 0.006594431703778584 |
| 1775 | Klhl23 | 0.006577436745654058 |
| 1649 | Cd33 | 0.006557029115996961 |
| 2039 | Cyp51 | 0.006548075707177992 |
| 2711 | Mtus1 | 0.0065411834772450776 |
| 142 | Ch25h | 0.006527095375600176 |
| 2384 | Ext1 | 0.006523582108370812 |
| 3228 | Cln8 | 0.006512995186742325 |
| 1944 | Cd80 | 0.006506529278027449 |
| 85 | Igfbp2 | 0.006484700410915375 |
| 96 | Ccn2 | 0.0064780091109562675 |
| 2764 | Cemip2 | 0.006472483581721643 |
| 1211 | Tnfrsf9 | 0.0064699915196171935 |
| 2464 | Lmo2 | 0.006425055184705984 |
| 2243 | Ly6e | 0.006405917285716625 |
| 2767 | Mcm3 | 0.006404738949920209 |
| 1155 | Hspb8 | 0.0063970771327578994 |
| 2235 | Susd2 | 0.006383079484653023 |
| 860 | Mgst1 | 0.006379598162300132 |
| 264 | Adm | 0.006378380817806548 |
| 830 | ENSRNOG00000019050 | 0.006369307901414753 |
| 2943 | Flnc | 0.006368702549226632 |
| 410 | Jam2 | 0.006361687570691081 |
| 1516 | Itgam | 0.006357810977410867 |
| 543 | Nabp1 | 0.00631814303442765 |
| 748 | Serpinb2 | 0.006312822189450623 |
| 3056 | Phlda2 | 0.006311056796758955 |
| 879 | Slco2b1 | 0.006229780274957481 |
| 2682 | Fli1 | 0.006202803653025215 |
| 1464 | Tfrc | 0.00619989430940169 |
| 1311 | S100b | 0.006185218660457053 |
| 2098 | P4ha2 | 0.006180682488433514 |
| 3177 | Zfp347 | 0.006165321676687887 |
| 1141 | Alox5ap | 0.00613853402067152 |
| 1110 | Gmnn | 0.0061314668140490785 |
| 1331 | Lmo1 | 0.006059587721021069 |
| 2092 | Mmp23 | 0.0060542844925902855 |
| 1250 | Mmd | 0.006048656190464927 |
| 1029 | Gjb2 | 0.006041293547252769 |
| 2263 | Cyth4 | 0.006040924098032508 |
| 328 | H1f5 | 0.006025860418052229 |
| 1358 | Bmper | 0.006018177219435902 |
| 499 | Csf1r | 0.006017242826318525 |
| 2878 | Gpr146 | 0.006000537882033856 |
| 2203 | Tnni2 | 0.005992229813825798 |
| 396 | Dnase1l3 | 0.005981926765227272 |
| 2188 | Cyrib | 0.005972974089544068 |
| 652 | Lgmn | 0.005969311933632536 |
| 1174 | Gdap1 | 0.005951731120637408 |
| 299 | Dpep1 | 0.005927919844829205 |
| 591 | Cyp1b1 | 0.005924587274257063 |
| 1437 | Chrna1 | 0.0058853477972965985 |
| 3034 | Nr2f1 | 0.005883978341286037 |
| 2836 | Tbc1d9 | 0.00587763435625176 |
| 810 | Mpzl1 | 0.005827872839935286 |
| 3098 | Tnfaip2 | 0.005826996566220432 |
| 2487 | Hells | 0.005825119085451367 |
| 2694 | Tst | 0.0058164203859363064 |
| 1825 | ENSRNOG00000070591 | 0.005808531347365338 |
| 1551 | Ntrk3 | 0.005779719240026197 |
| 2206 | Ldlr | 0.005777167983865582 |
| 1624 | Tnfrsf25 | 0.005764242104929759 |
| 53 | Obp3 | 0.005742835855630941 |
| 3187 | Zbtb2 | 0.005731131714312132 |
| 1911 | Cacnb3 | 0.005722824247742803 |
| 48 | Cryab | 0.0057100603507797484 |
| 1252 | Fam180a | 0.005708654838873275 |
| 3030 | Mcm7 | 0.00562767445032544 |
| 2924 | Tff1 | 0.005617831603138531 |
| 1618 | ENSRNOG00000004249 | 0.0056168612208856205 |
| 1379 | Serpinf1 | 0.00559799163422042 |
| 732 | Ms4a6bl.1 | 0.005567457701451209 |
| 23 | Myh11 | 0.005557664865194265 |
| 115 | Prg4 | 0.005551621664512483 |
| 3045 | Rspo1 | 0.005540561353766502 |
| 805 | Timp4 | 0.005516195601538147 |
| 963 | Fcgr2a.1 | 0.00551017069246934 |
| 1198 | Ehd3 | 0.005501014913181303 |
| 228 | Hist1h2an | 0.005423770994719419 |
| 262 | Adamts1 | 0.005423693385738334 |
| 836 | ENSRNOG00000062648 | 0.005416148589491757 |
| 2019 | Pcdhb9 | 0.005408114381944158 |
| 3064 | Ptk2b | 0.005394508459795291 |
| 1929 | Trib2 | 0.005379248773664875 |
| 1725 | AC097153.2 | 0.0053435583640794974 |
| 249 | Fcrl2 | 0.005300592856961181 |
| 1370 | Aspn | 0.005280475857113033 |
| 2187 | Batf | 0.005273088848594582 |
| 1135 | Tmem119 | 0.00522488823050842 |
| 76 | Ccl20 | 0.005209159504345789 |
| 2321 | Hcls1 | 0.005208384258188852 |
| 1200 | Tmcc3 | 0.005202259432286334 |
| 242 | Nr2f2 | 0.005191045056745325 |
| 2185 | Fjx1 | 0.005181774637728233 |
| 1408 | Ccl27 | 0.005172337764812962 |
| 2119 | Rps6ka1 | 0.0051572802975820555 |
| 1274 | Hhex | 0.005150869730492072 |
| 2552 | ENSRNOG00000066582 | 0.005145025853930818 |
| 423 | Spon1 | 0.005107476905130982 |
| 218 | Mmp11 | 0.00509052073340294 |
| 1575 | Ifitm10 | 0.005074145698251823 |
| 2641 | Niban2 | 0.005071502788782385 |
| 119 | Serpinb1a | 0.0050633489340143265 |
| 1726 | Rgs3 | 0.005050656390525035 |
| 704 | ENSRNOG00000006471 | 0.005041459667679534 |
| 170 | Ccl24 | 0.005038747829266069 |
| 1579 | Birc3 | 0.005007575119379944 |
| 2818 | Foxp2 | 0.0049933032596666685 |
| 2816 | Mertk | 0.00498882907091564 |
| 851 | ENSRNOG00000069031 | 0.004968080787683207 |
| 1922 | Uaca | 0.004967840830702585 |
| 1930 | Itpr1 | 0.00495268280761359 |
| 2397 | Wwp1 | 0.004950413535349569 |
| 74 | Ptn | 0.004943914428037729 |
| 408 | Sell | 0.004936289984048335 |
| 2921 | Rcsd1 | 0.004916513852115931 |
| 2072 | Aoah | 0.004912502617765977 |
| 309 | Rel | 0.004891793035605308 |
| 2779 | Deptor | 0.004881206197960109 |
| 2284 | Lrrc25 | 0.004870919449964069 |
| 188 | Actg2 | 0.004852957277876528 |
| 2987 | Mis18bp1 | 0.004850195894080311 |
| 292 | Itgb4 | 0.004847018000273694 |
| 1101 | ENSRNOG00000069640 | 0.004811895062275702 |
| 1788 | Mylpf | 0.004795313719764176 |
| 1113 | Gbp2 | 0.004789679691007574 |
| 1813 | Agrn | 0.0047770928425773535 |
| 3121 | Bcar1 | 0.004775670370436868 |
| 682 | Ccrl2 | 0.004775069884850442 |
| 3092 | Plekhm3 | 0.0047740788319230515 |
| 3090 | ENSRNOG00000070316 | 0.004760942840867742 |
| 1560 | Hck | 0.004748907575988412 |
| 2759 | Cd38 | 0.004746144710727174 |
| 1015 | Plekho1 | 0.004744772239135459 |
| 454 | Asgr2 | 0.004733035782478259 |
| 750 | ENSRNOG00000066750 | 0.004727305839680026 |
| 1398 | Dapp1 | 0.004721100065820747 |
| 971 | Gria2 | 0.004709708316359661 |
| 3046 | Epha4 | 0.0047089994672943 |
| 1057 | Cdkn1a | 0.0047008773410558355 |
| 2519 | Mcm6 | 0.00469983388443697 |
| 992 | Rasa4 | 0.0046908332026072055 |
| 441 | Tnfrsf18 | 0.004652059096873119 |
| 993 | ENSRNOG00000023122 | 0.004651263402778538 |
| 3094 | Adh7 | 0.0046274543926110665 |
| 3163 | Arg2 | 0.004620702269530287 |
| 1698 | ENSRNOG00000062837 | 0.004609437688581672 |
| 2547 | ENSRNOG00000064982 | 0.0045897009774992885 |
| 2993 | Lrrc8c | 0.004580550395939649 |
| 2177 | Ldhb | 0.0045584349475420215 |
| 1486 | Klf2 | 0.004553338037911692 |
| 2603 | Atad5 | 0.004548721629943257 |
| 708 | Slc28a2 | 0.004544255802777505 |
| 2977 | Crlf2 | 0.004525297865241912 |
| 1996 | Epsti1 | 0.004519619778892207 |
| 1884 | Smarcd3 | 0.004507957469999075 |
| 530 | Mpeg1 | 0.004493800790815674 |
| 784 | Ctla2a | 0.00446858460066786 |
| 150 | ENSRNOG00000067310 | 0.0044512496169388524 |
| 2329 | Dpep2 | 0.004448017978622192 |
| 1262 | Samsn1 | 0.004447378292949845 |
| 2532 | Smtn | 0.00443873171459838 |
| 986 | Sox4 | 0.004426497846962367 |
| 3158 | Slc39a10 | 0.004425506654460997 |
| 1593 | Batf3 | 0.004423368293561673 |
| 2514 | Haus6 | 0.00442101886930596 |
| 2796 | Kank1 | 0.004410634393475049 |
| 2404 | Lcp2 | 0.004407881640364137 |
| 2797 | Wnt5a | 0.004397709863553134 |
| 1484 | Fyb1 | 0.004396508586879142 |
| 1777 | P2ry13 | 0.004354262563788127 |
| 705 | Myo1b | 0.0043519390163023145 |
| 2345 | S100a3 | 0.004344800095553783 |
| 2234 | Irx5 | 0.00431658028241968 |
| 1677 | Fxyd6 | 0.00430755401622239 |
| 880 | F11r | 0.004303675999114525 |
| 2180 | Cd274 | 0.004298191035087447 |
| 1873 | Epgn | 0.004295237648448325 |
| 628 | Zfat | 0.00426461764299088 |
| 883 | Lipe | 0.004261042964452226 |
| 1490 | Rab20 | 0.004260103373209426 |
| 1617 | Gpr88 | 0.004246932547213032 |
| 3136 | Htr2b | 0.004233205154025023 |
| 559 | Ppt1 | 0.004192631745271828 |
| 457 | Stab1 | 0.004191933825307555 |
| 1805 | Prkcz | 0.0041859066075627714 |
| 2054 | Cxcl16 | 0.004173918360725644 |
| 1184 | Jam3 | 0.004173252792791821 |
| 306 | Il1rl1 | 0.004157696800962959 |
| 549 | Scimp | 0.004152611361021477 |
| 1413 | Col14a1 | 0.004145026683440547 |
| 1465 | Igfbp4 | 0.004136205307846115 |
| 2798 | Rad51 | 0.004132227896387678 |
| 2727 | Plk4 | 0.004127247707951356 |
| 859 | Plin2 | 0.004122060146426297 |
| 2676 | Plcxd2 | 0.0041161830530015205 |
| 2477 | Col6a5 | 0.00410839723462255 |
| 361 | Lmcd1 | 0.0040932597256757835 |
| 551 | Calca | 0.004092673031649969 |
| 779 | Tmem37 | 0.004087746188654448 |
| 2522 | U3.38 | 0.004086183186410948 |
| 1857 | Ccr10 | 0.004065889784703659 |
| 777 | Tcf15 | 0.004061880967284369 |
| 1646 | Lrrn3 | 0.0040571957276148785 |
| 265 | Klrd1 | 0.004030490326111577 |
| 2800 | Fkbp7 | 0.004019236474033566 |
| 2784 | Sim2 | 0.0040123324823031865 |
| 394 | Ccl9 | 0.003997644303141205 |
| 282 | Ifit1bl | 0.003993574004376482 |
| 1218 | Ctse | 0.0039888997359477565 |
| 398 | Slc9a3r2 | 0.003978474063018782 |
| 2462 | Nexmif | 0.003973669761578128 |
| 3103 | Rab27a | 0.003934098670955351 |
| 553 | ENSRNOG00000070011 | 0.00393234458674963 |
| 1604 | Plcb4 | 0.003929016859770236 |
| 848 | B3gnt2 | 0.003927693475382277 |
| 864 | Csn1s2b | 0.0039002409211709456 |
| 1396 | RGD1563888 | 0.0038958479325277473 |
| 1860 | Ceacam1 | 0.003892734082105682 |
| 133 | Sele | 0.0038851729186863376 |
| 330 | Rasd1 | 0.0038754465416702515 |
| 2416 | Cyp2d3 | 0.0038596035507393748 |
| 546 | Palmd | 0.003858325985396125 |
| 815 | Nfkbid | 0.0038497725863022338 |
| 1529 | Krt5 | 0.003833526591030786 |
| 841 | Ccn3 | 0.0038153981537188807 |
| 384 | Vwa1 | 0.00380574379087775 |
| 1896 | P2ry12 | 0.0037966495776227092 |
| 964 | Angptl8 | 0.003795097379702449 |
| 448 | Akap2 | 0.003785790473628285 |
| 760 | Rac2 | 0.0037852125999341124 |
| 3000 | Angpt1 | 0.003759694629885857 |
| 1916 | Sectm1a | 0.0037417138133036404 |
| 2328 | Irx2 | 0.0037407642287352622 |
| 1745 | Stard8 | 0.003714966905891734 |
| 2457 | AC128859.3 | 0.0037097859359966298 |
| 525 | Il2rb | 0.0036535718890533895 |
| 103 | Mki67 | 0.0036520129607020427 |
| 812 | Ctss | 0.003643449895010198 |
| 334 | Erg | 0.0036295482531647654 |
| 2423 | Rcn3 | 0.0036084985328499993 |
| 1582 | Klrk1 | 0.0036051586503982025 |
| 912 | Hmcn1 | 0.0036041767320612603 |
| 2595 | Gfra2 | 0.003595115838097451 |
| 3225 | Nt5dc2 | 0.003555842161457399 |
| 2132 | Cldnd1 | 0.0035519634132028686 |
| 1460 | Gbp5 | 0.0035454287268198645 |
| 2120 | ENSRNOG00000063634 | 0.0035406830957363557 |
| 2649 | Phgdh | 0.0035334986824791855 |
| 1778 | Gna15 | 0.0035271954882145308 |
| 2372 | Entpd1 | 0.0035199862566174705 |
| 1762 | ENSRNOG00000061530 | 0.003496251555597781 |
| 1345 | Maff | 0.003491846168288887 |
| 2947 | ENSRNOG00000066286 | 0.003483601696814679 |
| 1356 | Slc16a3 | 0.0034743353751635593 |
| 2418 | Nfam1 | 0.0034702329340672937 |
| 1534 | Trarg1 | 0.00346268387464568 |
| 2611 | Ggh | 0.003448092166563651 |
| 1648 | Klhl41 | 0.0034458801354978613 |
| 2572 | Rtn4rl1 | 0.0034206052296381403 |
| 417 | Atp1b2 | 0.0034202114986603277 |
| 1462 | ENSRNOG00000018960 | 0.00341858425130473 |
| 2041 | Hlx | 0.0034075083784870785 |
| 1165 | Nptx1 | 0.0033901259772277886 |
| 1436 | Clec4a1 | 0.003389204294458296 |
| 3044 | Slc1a7 | 0.0033811724920705397 |
| 177 | Dnaaf2 | 0.0033723539517624555 |
| 146 | Tmem45b | 0.003343665823924781 |
| 944 | Fxyd7 | 0.003338209937772425 |
| 2718 | Cnn2 | 0.003333908199053791 |
| 2306 | Tifab | 0.0033332878248084888 |
| 2056 | Dkk3 | 0.0033302424229704475 |
| 1483 | Sqle | 0.0033030086275581344 |
| 951 | Ect2 | 0.0033013088540151713 |
| 1811 | Cish | 0.0032888301238774395 |
| 2454 | Ppp1r3b | 0.0032655388972919466 |
| 2558 | Pcdh10 | 0.003251775272975147 |
| 1191 | Fcgr1a | 0.00324948250640102 |
| 1259 | Apobec1 | 0.0032302764603025728 |
| 489 | Klre1 | 0.003217459435897577 |
| 2949 | Prkcb | 0.0032021408129409767 |
| 554 | S1pr1 | 0.0031999893027702736 |
| 2085 | ENSRNOG00000028899 | 0.003180739557597374 |
| 1476 | Btla | 0.003177486372363804 |
| 1336 | Dok2 | 0.003173337711293665 |
| 1080 | Creg1 | 0.003171854444062425 |
| 906 | Itgb7 | 0.003167591098143077 |
| 524 | Hey1 | 0.0031517742582549716 |
| 2862 | N4bp2l1 | 0.0031484150319977013 |
| 1196 | Ier5 | 0.003133803376497625 |
| 276 | ENSRNOG00000068953 | 0.0031225696282449595 |
| 3159 | Cyp2c11 | 0.003106588305071169 |
| 362 | Spc24 | 0.003103492359308961 |
| 1709 | Arhgap9 | 0.0031030179106127324 |
| 239 | Rrad | 0.0031014269099914546 |
| 425 | Ptprr | 0.0030998996971231788 |
| 709 | Csrp1 | 0.0030919688976905825 |
| 124 | Hcar2 | 0.003066655292904942 |
| 2766 | Arhgap15 | 0.0030149995605913186 |
| 1404 | Cdc42ep3 | 0.003014702871529624 |
| 668 | AABR07006275.1 | 0.003006521015537727 |
| 34 | Cytl1 | 0.0030031298070564367 |
| 3195 | Snorc | 0.0029989287776469487 |
| 509 | Nfkbiz | 0.002992917880660165 |
| 3025 | Filip1 | 0.0029841880650889386 |
| 643 | Bcr | 0.002981575253824735 |
| 1282 | Evl | 0.0029789751103905075 |
| 2833 | RGD1566134 | 0.002957537205434074 |
| 824 | Naaa | 0.002952174100545916 |
| 569 | Cd93 | 0.0029406424541637334 |
| 1497 | Siglec5 | 0.0029385538735459396 |
| 1918 | Irx1 | 0.002926849389122477 |
| 1070 | Adam8 | 0.002924746264937112 |
| 855 | Gvin1 | 0.0029177054141871373 |
| 2032 | Cd151 | 0.0029149590970536324 |
| 1325 | Slamf9 | 0.002902058083246687 |
| 1718 | ENSRNOG00000070027 | 0.002894407266229285 |
| 2382 | Irs1 | 0.0028880964304632242 |
| 350 | Prc1 | 0.0028813717555440403 |
| 2063 | Atp1a3 | 0.002878677892766517 |
| 2722 | Daam1 | 0.0028784247088783966 |
| 601 | Cav2 | 0.0028711040404150808 |
| 308 | Cd2 | 0.0028660159694742704 |
| 3114 | P2ry1 | 0.0028595769669284184 |
| 1217 | ENSRNOG00000011513 | 0.0028574510060982317 |
| 1869 | Kctd4 | 0.0028573770159224933 |
| 2388 | Pcdh17 | 0.0028422574558092766 |
| 2760 | Gramd2b | 0.002839689642732209 |
| 1824 | Tpd52 | 0.002837164609530013 |
| 2011 | Prr7 | 0.0028351177080926633 |
| 619 | Runx2 | 0.002834748878576131 |
| 2237 | Pianp | 0.0028182305341706935 |
| 3043 | Myom3 | 0.0028171951427321423 |
| 1882 | Ctsk | 0.0028104477063810125 |
| 2583 | Mex3b | 0.002807767926532337 |
| 2607 | ENSRNOG00000043342 | 0.002804677095742241 |
| 2151 | Clec11a | 0.0028002764814333008 |
| 867 | Pde2a | 0.0027878454075058214 |
| 1187 | Tspan13 | 0.002787608704257481 |
| 1877 | Cd53 | 0.0027857313251309244 |
| 60 | Myl9 | 0.0027835787081846464 |
| 98 | Smoc2 | 0.0027774412175367033 |
| 1535 | Alox5 | 0.0027760846874873746 |
| 2142 | Fermt3 | 0.002771818886186975 |
| 33 | Aqp1 | 0.0027701031085761146 |
| 783 | ENSRNOG00000046548 | 0.002768824743392621 |
| 985 | Cd96 | 0.0027575387585738686 |
| 1333 | Icam2 | 0.002755414952655593 |
| 2495 | Abcg1 | 0.0027471699766726185 |
| 2824 | Myo1f | 0.002746015984429949 |
| 903 | Cd247 | 0.0027384893728035127 |
| 2049 | Cyb561 | 0.0027358421658877675 |
| 2967 | Krt17 | 0.0027336271527583487 |
| 59 | Sfrp2 | 0.002708089656187145 |
| 1572 | Inhbb | 0.002708002032704922 |
| 501 | Alkal2 | 0.002690008888766459 |
| 2509 | Zfp608 | 0.002686611414805345 |
| 2698 | Mpp2 | 0.0026813738862385117 |
| 980 | Sox7 | 0.002681047958047405 |
| 1016 | Ctsz | 0.0026787221280340488 |
| 1322 | Nat8f3 | 0.0026707366638125457 |
| 2059 | Khdrbs3 | 0.0026617492057468732 |
| 936 | Clec4a3 | 0.0026562925429734928 |
| 807 | Rassf4 | 0.0026551318593479294 |
| 79 | Hp | 0.0026479310484413213 |
| 2031 | Lta4h | 0.0026478804131504175 |
| 2785 | Tacc3 | 0.0026230021162861284 |
| 488 | Cenpw | 0.00262207939559405 |
| 44 | Gzmm | 0.0026059040414499078 |
| 216 | Rgs16 | 0.002571124345496467 |
| 1037 | Alpl | 0.0025592470110331923 |
| 397 | Napsa | 0.0025547257218729415 |
| 2830 | Slc8a1 | 0.002538382454404973 |
| 1904 | Clec12a | 0.0025339444424559293 |
| 2940 | ENSRNOG00000068602 | 0.0024954938749672108 |
| 2553 | Myoz1 | 0.0024655103359184933 |
| 1227 | Padi2 | 0.002444509493485121 |
| 1626 | Igsf6 | 0.0024408021335664714 |
| 1665 | Igsf9 | 0.0024404431794125873 |
| 1701 | Klra2 | 0.002437276204937904 |
| 3226 | ENSRNOG00000065676 | 0.0024370270895247006 |
| 2750 | Plod2 | 0.002434137440277768 |
| 1943 | ENSRNOG00000065876 | 0.0024284053640921018 |
| 3048 | ENSRNOG00000068991 | 0.0024262474709690314 |
| 1742 | Fmo3 | 0.002422848002379139 |
| 1759 | ENSRNOG00000064487 | 0.002422132362569818 |
| 674 | Emb | 0.0024180516776934535 |
| 1477 | Mlf1 | 0.002417117930189553 |
| 3224 | Shroom4 | 0.0024158503897333244 |
| 3086 | Gatm | 0.0024098486605774122 |
| 2353 | Cdkn2d | 0.0024081250124482357 |
| 343 | Gpr171 | 0.002407752900001686 |
| 1897 | Rad51b | 0.0023968871017472373 |
| 1978 | Gmfg | 0.0023829697964760817 |
| 3165 | Col27a1 | 0.0023817892247679252 |
| 259 | Septin4 | 0.002380879453366523 |
| 9 | Retnlg | 0.0023674621008909326 |
| 2511 | Tiam1 | 0.0023641135461510776 |
| 3191 | RGD1559575 | 0.002353314933750572 |
| 1679 | Septin6 | 0.0023491332992889996 |
| 787 | Tent5b | 0.0023428328339792736 |
| 1685 | Cidea | 0.0023316636019330575 |
| 2804 | ENSRNOG00000067240 | 0.002331129777119837 |
| 378 | Fcmr | 0.002330447316450831 |
| 1467 | Unc119 | 0.002325439357107822 |
| 514 | Chrne | 0.0023200041359473906 |
| 989 | Sema4d | 0.0023188621649606377 |
| 1670 | Hmmr | 0.002318074459771052 |
| 1240 | Ca3 | 0.002317427293472302 |
| 12 | Tagln | 0.002303150214387897 |
| 3134 | Irs3 | 0.002298484079534405 |
| 1994 | Ptpro | 0.00229700655682302 |
| 684 | Hdc | 0.002284433600349022 |
| 1339 | Tnfsf18 | 0.002284021024658566 |
| 131 | Spp1 | 0.002281773223922135 |
| 2866 | Epdr1 | 0.0022726984322852285 |
| 2945 | Sap30 | 0.0022714144237293106 |
| 900 | Siglec1 | 0.0022607256311258503 |
| 400 | Tnf | 0.0022576288945943086 |
| 749 | Cytip | 0.0022531246440462985 |
| 151 | Fmod | 0.002251932099277404 |
| 2559 | Tshz2 | 0.0022483329216152984 |
| 725 | Arc | 0.00223460025366758 |
| 2408 | Cacna1g | 0.0022267080721444935 |
| 207 | Vwf | 0.0022232315111924607 |
| 2450 | Rcn1 | 0.002216670933178351 |
| 2262 | Stk17b | 0.0022063652462994687 |
| 2864 | ENSRNOG00000003748 | 0.0022031089340914835 |
| 962 | Themis2 | 0.0021987593162531398 |
| 1613 | Sphk1 | 0.0021861731309237393 |
| 1206 | Apoe | 0.0021844892608767893 |
| 40 | Clu | 0.002175537265305105 |
| 1427 | Kank3 | 0.0021647169575552197 |
| 2709 | Yes1 | 0.0021590377890492547 |
| 2781 | Slc7a5 | 0.0021570680725156114 |
| 2344 | Slc6a9 | 0.0021544486862374998 |
| 528 | Pcsk1n | 0.002131412414075534 |
| 178 | Nppc | 0.00213025164760599 |
| 2194 | Rcan1 | 0.0021135340520381544 |
| 843 | ENSRNOG00000037661 | 0.002104142574956994 |
| 270 | Mcemp1 | 0.002103718249051291 |
| 138 | Id1 | 0.0021016624006699475 |
| 114 | Mmp12 | 0.0021002762907096958 |
| 1461 | Hspb6 | 0.0020998192211053 |
| 791 | Esam | 0.002089993940187368 |
| 1982 | Bcl6b | 0.0020884147677234757 |
| 3199 | Eepd1 | 0.002085191232662026 |
| 3113 | Fzd6 | 0.00208504026288498 |
| 486 | Gucy1a1 | 0.002076921514265227 |
| 1830 | Pim2 | 0.0020689784704005813 |
| 1127 | Ms4a6a | 0.002067113103663767 |
| 3147 | Arhgap45 | 0.002065846475288696 |
| 460 | Bex1 | 0.0020600711930578207 |
| 244 | Ctsw | 0.0020414451141404716 |
| 2915 | Fgd2 | 0.002032629913011258 |
| 3171 | Gpd1 | 0.002028729673407982 |
| 250 | ENSRNOG00000062811 | 0.002020431399380907 |
| 55 | Acta2 | 0.0020199358813938347 |
| 50 | Stmn2 | 0.0020171046964090503 |
| 2774 | Plekhg5 | 0.0020100005408010832 |
| 320 | AC141102.1 | 0.0020094623459840327 |
| 2782 | Myo1d | 0.0019945515741369947 |
| 1902 | Lyl1 | 0.0019871914739173443 |
| 666 | Flt3 | 0.001981765138423734 |
| 1768 | Spint1 | 0.0019814773668084024 |
| 1942 | Cd86 | 0.0019797070824921718 |
| 2480 | Traf4 | 0.0019710912506091293 |
| 919 | Igfbp6 | 0.001963411753000456 |
| 2762 | Shox2 | 0.0019430727750691962 |
| 2080 | Cenpm | 0.0019393038051470518 |
| 377 | Cox4i2 | 0.0019311968797920817 |
| 2962 | Slc22a1 | 0.0019235817352738094 |
| 2040 | Evi2b | 0.0019200924331120074 |
| 1389 | Me2 | 0.0019196505014696542 |
| 2481 | Ptpn18 | 0.00191815511710736 |
| 117 | ENSRNOG00000070909 | 0.0019078179186249986 |
| 1199 | Abi3 | 0.0019038862391858036 |
| 2712 | Hist2h3c2.5 | 0.0019022890116112258 |
| 285 | Slamf7 | 0.001892433777197927 |
| 818 | Rnf152 | 0.0018916436847779222 |
| 904 | Lax1 | 0.0018880049696238421 |
| 311 | Ptprcap | 0.0018849032415873027 |
| 734 | Spns2 | 0.0018784986744674876 |
| 2017 | Cip2a | 0.0018718150615898617 |
| 346 | ENSRNOG00000002820 | 0.0018684005127051068 |
| 540 | Net1 | 0.0018599622127268706 |
| 2402 | Acap1 | 0.0018585711937137536 |
| 2548 | Skap2 | 0.0018571390315402652 |
| 1126 | Bricd5 | 0.0018560310215029957 |
| 1391 | Pygm | 0.0018543234497434623 |
| 2303 | Il10ra | 0.0018471522718288727 |
| 2803 | Csf2 | 0.0018435909241979421 |
| 1511 | Tekt3 | 0.0018289694734613465 |
| 1812 | Ptpn6 | 0.0018253208256981247 |
| 2551 | Plxnc1 | 0.0018233556775602438 |
| 1310 | Galnt18 | 0.0018211361206603993 |
| 548 | Mest | 0.0018194760394769703 |
| 3 | Plvap | 0.0018181646479623085 |
| 1758 | Ddr2 | 0.0018152007736754957 |
| 1018 | Aurkb | 0.0018115473150889043 |
| 8 | Il17a | 0.001810485321080342 |
| 2176 | Apol3 | 0.0018103426587782357 |
| 97 | Il17f | 0.0017990698526805012 |
| 2673 | Satb1 | 0.0017943555765946317 |
| 1422 | P2ry10 | 0.0017827334634054608 |
| 2667 | Cmklr1 | 0.0017757520756252415 |
| 241 | Col15a1 | 0.0017716504588837275 |
| 463 | Mybpc1 | 0.001770520794895281 |
| 934 | Hrc | 0.0017588856766168402 |
| 1313 | Gpr65 | 0.0017489969521250963 |
| 2365 | Adarb1 | 0.0017386648473208767 |
| 3036 | Nos3 | 0.0017324118971204364 |
| 1797 | Mgat3 | 0.0017310226842739832 |
| 1661 | RGD1305184 | 0.001728085390397042 |
| 1161 | Tbc1d4 | 0.0017242847829547622 |
| 2659 | Otulinl | 0.0017234887052930067 |
| 1730 | Selplg | 0.0017186651074762118 |
| 2891 | Tnfrsf22 | 0.0017151461629885865 |
| 2147 | Prrx1 | 0.0016989719150150415 |
| 1543 | Rbpms | 0.0016956529252327118 |
| 3189 | Ccdc141 | 0.0016936096716843227 |
| 322 | AABR07000398.1 | 0.0016911853529743709 |
| 1756 | ENSRNOG00000063741 | 0.0016890223979454866 |
| 1142 | Plscr2 | 0.001685209154903374 |
| 2665 | Fry | 0.0016850013433091708 |
| 240 | Mylk | 0.001679880953270643 |
| 2396 | Tjp1 | 0.0016792430956811678 |
| 3211 | Trim14 | 0.0016775429578595796 |
| 811 | Fmo2 | 0.0016678993031596246 |
| 2684 | Adcy4 | 0.0016564938996976772 |
| 1030 | ENSRNOG00000062844 | 0.0016536631141477384 |
| 2100 | Emid1 | 0.0016529746558472456 |
| 1472 | F2rl1 | 0.001646438238983332 |
| 2248 | Fzd8 | 0.0016434073120953777 |
| 2565 | Nup210 | 0.001634837607916132 |
| 2929 | Srcin1 | 0.0016347233842552529 |
| 52 | Rnase6 | 0.0016310138384911604 |
| 1277 | Pgf | 0.001619185665297306 |
| 1838 | Iqgap2 | 0.0016170312819929473 |
| 1491 | Popdc2 | 0.0016065798260956345 |
| 3022 | Crym | 0.001606481642140058 |
| 2285 | Wee1 | 0.0016026102109468366 |
| 1148 | Wnt16 | 0.0016016861723645115 |
| 1019 | Csf3r | 0.0015986894628640016 |
| 2644 | Ccdc34 | 0.0015953539677846032 |
| 2122 | ENSRNOG00000060241 | 0.0015924810695848298 |
| 816 | Matk | 0.0015921073214991179 |
| 1095 | Tmem176b | 0.0015861604393422864 |
| 1995 | Hist2h3c2.4 | 0.0015690020772125058 |
| 1564 | Pmel | 0.0015618104459308078 |
| 585 | Tph1 | 0.001556605855391567 |
| 827 | Fscn1 | 0.0015493400570253234 |
| 2433 | Rerg | 0.0015334406316290592 |
| 3213 | Ppp1r12c | 0.0015260865213793133 |
| 719 | Icos | 0.001513677646559688 |
| 129 | Fcgr3a | 0.001513113992837678 |
| 1239 | Scin | 0.0015111988789557709 |
| 1140 | Lama5 | 0.0015014574412553756 |
| 2283 | Ikzf2 | 0.001500832370942146 |
| 326 | Il2ra | 0.0014924933491199453 |
| 1790 | Gpr176 | 0.0014773120361166485 |
| 1293 | ENSRNOG00000038686 | 0.001474916431057656 |
| 3055 | Kif18a | 0.0014747385600961055 |
| 64 | Cd209f | 0.0014728497776848994 |
| 941 | Il2rg | 0.0014709070806831846 |
| 1320 | Mefv | 0.001467069608852221 |
| 625 | Racgap1 | 0.0014448349377947447 |
| 224 | Apold1 | 0.0014429416914380332 |
| 970 | Pcsk1 | 0.0014372022664153735 |
| 1619 | Syngr1 | 0.001433833157079121 |
| 1608 | Aldh1a2 | 0.0014317306002996218 |
| 2586 | Kcnb1 | 0.0014231613982581457 |
| 272 | Nusap1 | 0.0014177809913985216 |
| 2275 | Pstpip1 | 0.001417655610450332 |
| 374 | Tnfaip3 | 0.001415571315747156 |
| 2825 | Prdm16 | 0.0014147574977020172 |
| 2443 | Dok3 | 0.0013904339389541889 |
| 1563 | Ncf1 | 0.001389065800518606 |
| 7 | Fcnb | 0.0013885458885381811 |
| 1364 | Slc45a3 | 0.0013794739385194963 |
| 1124 | Nes | 0.0013701524649793672 |
| 2871 | Strip2 | 0.001367021666790374 |
| 2045 | Il18rap | 0.0013535025320055428 |
| 368 | AABR07005844.1 | 0.0013501686405246512 |
| 1664 | Nbl1 | 0.001348319147599328 |
| 1806 | Slc44a2 | 0.0013312919610236505 |
| 2858 | Hoxd10 | 0.0013264452110366616 |
| 649 | Ccnb1 | 0.0013236202282371917 |
| 794 | ENSRNOG00000013727 | 0.0013205548174282811 |
| 1061 | Enpep | 0.0013203372955141019 |
| 496 | Col13a1 | 0.0013192248011274594 |
| 2790 | Sfn | 0.0013188807739765874 |
| 337 | Adgrg1 | 0.0013160964590058534 |
| 729 | Cenpe | 0.0013124016516378708 |
| 3096 | Speg | 0.0013106877271744106 |
| 2979 | LRRTM1 | 0.0013052409242884592 |
| 607 | Ramp3 | 0.001291716650263563 |
| 1743 | AABR07044631.2 | 0.0012911391552723086 |
| 1348 | Ebi3 | 0.0012887394919237718 |
| 741 | RT1-DMb | 0.0012873361904236363 |
| 2483 | Agxt | 0.001281545446970232 |
| 2951 | B3galt2 | 0.001280105082640236 |
| 2823 | Plppr5 | 0.0012775864084274753 |
| 722 | Hcst | 0.0012708987274689164 |
| 2323 | Fam171b | 0.0012707124163188008 |
| 2554 | ENSRNOG00000064143 | 0.001266673731135344 |
| 765 | Ccr5 | 0.0012597957336068467 |
| 277 | Cd3e | 0.0012594807584732205 |
| 632 | Igf2 | 0.0012530343077336347 |
| 3190 | Prr29 | 0.0012477288753838705 |
| 3019 | Pik3ap1 | 0.001242443651392483 |
| 375 | Nrarp | 0.0012395473309731136 |
| 88 | Cck | 0.0012359042430045313 |
| 1043 | ENSRNOG00000008057 | 0.0012318899137991194 |
| 614 | Lck | 0.0012274046309967228 |
| 3058 | P2rx3 | 0.00122588708054194 |
| 120 | Pglyrp1 | 0.0012208893140215546 |
| 1682 | Masp1 | 0.0012177162117311454 |
| 2678 | Ankrd37 | 0.0012138270593723029 |
| 1647 | Slc10a6 | 0.0012126104721768877 |
| 1546 | Nrn1 | 0.0012116408057208494 |
| 3009 | Cldn22 | 0.0012099494004677632 |
| 1984 | Ggta1 | 0.0012076646804280913 |
| 185 | Lyz2 | 0.0012071932094429706 |
| 348 | S100a5 | 0.0012054660077958962 |
| 1728 | Bub1 | 0.001195049612743824 |
| 2373 | Relt | 0.0011878738048989193 |
| 195 | Cdkn3 | 0.0011803289489814679 |
| 1241 | Cdca2 | 0.0011787292261590796 |
| 2806 | Ehf | 0.0011757729458536444 |
| 517 | Birc5 | 0.0011726620977230572 |
| 2928 | Tnfrsf26 | 0.0011707390507571785 |
| 792 | Lag3 | 0.001167911798900815 |
| 687 | Fcgr2b | 0.0011666651989159058 |
| 189 | Sfrp1 | 0.001159300209624288 |
| 1757 | ENSRNOG00000068515 | 0.001154684111970609 |
| 43 | Ube2c | 0.001149638501843896 |
| 2469 | Enho | 0.001147650993469202 |
| 1678 | Gprc5c | 0.0011471846155355913 |
| 1724 | ENSRNOG00000010454 | 0.0011449707775149681 |
| 1729 | Tmem38a | 0.001141429623378033 |
| 1630 | Ppp1r3c | 0.001116897920655936 |
| 411 | Oas1k.1 | 0.0011165311240669833 |
| 2783 | Foxc2 | 0.0011122635102681646 |
| 211 | Rrm2 | 0.0011106378083878287 |
| 1948 | Kcna3 | 0.0011092774868602423 |
| 2935 | Nr1h3 | 0.0011046076390556013 |
| 41 | Slpi | 0.0011030521042922365 |
| 2006 | Jph2 | 0.0010987355465565678 |
| 2892 | Elk3 | 0.0010981943834847332 |
| 623 | Il23a | 0.0010951037414253556 |
| 338 | Chchd10 | 0.0010950986010488527 |
| 414 | ENSRNOG00000069878 | 0.0010907163015883312 |
| 713 | Ezr | 0.00108887996583133 |
| 1502 | Stra8 | 0.0010886370529592545 |
| 360 | Acp5 | 0.0010857961022709246 |
| 3203 | Tlr5 | 0.0010843197789378617 |
| 2385 | Syt15 | 0.0010803149531629101 |
| 1919 | Crmp1 | 0.0010787613003979575 |
| 401 | Adgrf5 | 0.0010779551631784467 |
| 184 | RT1-Bb | 0.0010751252667001124 |
| 1740 | Lratd2 | 0.0010715131431605416 |
| 291 | Art2b | 0.0010711430683511027 |
| 465 | Ager | 0.0010646628453460077 |
| 1871 | ENSRNOG00000025200 | 0.0010544029245379985 |
| 1009 | Cd5 | 0.0010489287904919453 |
| 100 | Hba-a3 | 0.0010462415015807486 |
| 662 | Epas1 | 0.0010357545777547948 |
| 1571 | RGD1563400 | 0.001029471172596929 |
| 1352 | Stc2 | 0.0010278250224716756 |
| 1343 | Lilrb3l | 0.0010247185739721216 |
| 1111 | Cyb5r2 | 0.0010232855465390238 |
| 3232 | Dek | 0.0010192006689955695 |
| 1400 | ENSRNOG00000067110 | 0.0010186483813120627 |
| 518 | Blnk | 0.0010159570531200538 |
| 825 | Cesl1.1 | 0.0010116211884146945 |
| 599 | Tbx21 | 0.001011042273748744 |
| 2955 | Sox15 | 0.0010091980598021808 |
| 1247 | Dlgap5 | 0.0010072748320092404 |
| 1964 | Crispld1 | 0.00100562309238586 |
| 778 | Cited2 | 0.0010053131391608019 |
| 101 | Kdr | 0.0009909959681680173 |
| 15 | Rbp7 | 0.000984297983626239 |
| 1429 | Cenph | 0.0009824647511933236 |
| 1253 | ENSRNOG00000068899 | 0.0009768883165708652 |
| 1194 | Rnase1l2 | 0.0009750656538640666 |
| 2927 | ENSRNOG00000052407 | 0.0009747566917886815 |
| 644 | Sh2d2a | 0.0009744828945670675 |
| 2305 | Slc15a3 | 0.0009724816535350721 |
| 1792 | Atp2a3 | 0.0009691921857270715 |
| 590 | Actn1 | 0.000965695021050407 |
| 135 | Scarb1 | 0.000956072543480917 |
| 1278 | Trem1 | 0.0009512257181065983 |
| 1349 | Ptprf | 0.0009407442570227786 |
| 1180 | Rasl11b | 0.0009389992559891415 |
| 391 | Gdf10 | 0.0009376583798652645 |
| 2409 | Pkp4 | 0.0009356620833035588 |
| 1849 | Enpp2 | 0.0009197759467922698 |
| 983 | Fpr3 | 0.0009169003324923156 |
| 1245 | Ecscr | 0.0009141915455682563 |
| 1654 | Rasgef1b | 0.0009135803479421451 |
| 1881 | Cd82 | 0.0009128719695738938 |
| 1975 | Hoxb7 | 0.000912114977354728 |
| 436 | Faslg | 0.0009098700067685908 |
| 442 | Lef1 | 0.0009060601764736692 |
| 1802 | Myo5c | 0.0009009249801273665 |
| 225 | Cdh5 | 0.0008966418529259947 |
| 458 | Angpt2 | 0.0008874757868033098 |
| 2534 | ENSRNOG00000016978 | 0.0008850899787696218 |
| 107 | Rgs5 | 0.0008752482319300495 |
| 1817 | Pde4b | 0.0008750201830898813 |
| 1821 | Bin2 | 0.0008648843397839412 |
| 2252 | Cenpk | 0.0008605332799862012 |
| 2367 | Incenp | 0.0008585388464655013 |
| 220 | Fcna | 0.0008540077574681086 |
| 2008 | Itga4 | 0.0008496653664316518 |
| 1411 | Il21r | 0.0008477390066564134 |
| 2118 | Jaml | 0.000847555280938079 |
| 969 | RGD1565355 | 0.0008433878648880293 |
| 579 | Lat | 0.0008376021877121882 |
| 2036 | Ccne1 | 0.0008318448691154079 |
| 1810 | Csrp3 | 0.0008295396136766877 |
| 1209 | Ntsr1 | 0.0008213879583692428 |
| 2539 | Prkch | 0.0008198462700525148 |
| 740 | Septin1 | 0.0008186024878502893 |
| 1656 | Kcnj2 | 0.000818580737846966 |
| 1055 | Ppp1r1a | 0.0008182262230339097 |
| 1287 | Itga1 | 0.0008098176537986951 |
| 3145 | ENSRNOG00000064329 | 0.0008064650456486571 |
| 1296 | Dut | 0.0008064555805351451 |
| 1138 | Tbata | 0.0008047173395223657 |
| 2857 | Cyp4f18 | 0.0008029300611504121 |
| 2279 | F2r | 0.0007975416238772425 |
| 267 | Cdca3 | 0.0007947038914631218 |
| 1132 | Plk1 | 0.0007903382868537413 |
| 894 | Ccr3 | 0.0007894292101089744 |
| 758 | Cenpa | 0.0007867475265848637 |
| 1550 | Rhoh | 0.0007858619351876081 |
| 1752 | Bex4 | 0.0007827127338185997 |
| 2948 | Serinc2 | 0.0007800884075665579 |
| 1363 | Tnfaip8l2 | 0.0007794604714464011 |
| 2808 | Fstl3 | 0.0007736322748271014 |
| 538 | ENSRNOG00000066615 | 0.0007729644466611875 |
| 3067 | Arhgap11a | 0.0007726781092842795 |
| 415 | Gprc5d | 0.0007647684705273893 |
| 4 | Cma1 | 0.000764487737353632 |
| 1233 | Chac1 | 0.0007618947693833285 |
| 2549 | Syk | 0.0007616421163302738 |
| 2934 | AABR07029272.1 | 0.0007568271188055306 |
| 1299 | Tnfsf13 | 0.0007564662071940663 |
| 2957 | Susd3 | 0.0007548564002575833 |
| 1475 | Sdc1 | 0.0007521591940164045 |
| 1226 | Arhgef7 | 0.0007506431439064955 |
| 153 | Egfl7 | 0.0007473400141080966 |
| 562 | Bcl11b | 0.0007446438715730682 |
| 3140 | Cldn19 | 0.00074372437127032 |
| 967 | Tk1 | 0.0007432031652239858 |
| 2461 | RGD1307182 | 0.000743044940955078 |
| 1382 | Cpxm1 | 0.0007428518115219337 |
| 2241 | Tnnt1 | 0.0007425467472828609 |
| 2057 | Wdfy4 | 0.0007408845468090862 |
| 2755 | Dcx | 0.0007394129660873096 |
| 1143 | Hpgds | 0.0007363811647291803 |
| 61 | Nkg7 | 0.0007351422595104126 |
| 2592 | Rhof | 0.0007338207365115419 |
| 582 | Nppb | 0.0007335545056197929 |
| 2997 | Xirp2 | 0.0007334019468145298 |
| 1671 | Cdc6 | 0.000727116436998353 |
| 1265 | Tie1 | 0.0007225717005543441 |
| 1627 | Cldn23 | 0.0007210685305233582 |
| 2980 | Rapgef5 | 0.0007206373130972184 |
| 155 | Pclaf | 0.0007172235894963889 |
| 116 | Sbsn | 0.0007155215277001852 |
| 2739 | Dusp14 | 0.0007105111206712189 |
| 235 | AABR07017902.1 | 0.0007101868700208337 |
| 2317 | AABR07025140.1 | 0.0007048587761559542 |
| 1086 | Nap1l3 | 0.000699774661230025 |
| 1388 | Ncaph | 0.0006994555967551507 |
| 2170 | Zfp296 | 0.0006989799070308511 |
| 1234 | Spry4 | 0.000696901728781553 |
| 393 | Pttg1 | 0.0006935940061357478 |
| 2617 | ENSRNOG00000070630 | 0.0006864580189392762 |
| 1846 | P2ry6 | 0.0006864329313473097 |
| 1386 | Arl11 | 0.0006800489751460138 |
| 1190 | Aqp5 | 0.0006774751028033124 |
| 1635 | ENSRNOG00000067312 | 0.0006762960570018074 |
| 865 | Lilrb3 | 0.0006686776648229194 |
| 1500 | Lgals7 | 0.0006682146692006162 |
| 2089 | Hist2h3c2.3 | 0.0006635292726676524 |
| 2688 | Pde1b | 0.0006608527666099267 |
| 2141 | Ttc21a | 0.0006536657041070367 |
| 863 | Tnc | 0.0006516733854883493 |
| 1075 | Il1f10 | 0.000650884498064049 |
| 1751 | Evi2a | 0.0006456424353726009 |
| 922 | Bglap | 0.0006451234661329637 |
| 385 | Pmaip1 | 0.0006417871521773734 |
| 2145 | Sash3 | 0.0006413732531275509 |
| 932 | Cyp4b1 | 0.0006410672092162127 |
| 472 | Tpx2 | 0.0006409990039661934 |
| 2403 | ENSRNOG00000063814 | 0.00063700585628555 |
| 1112 | Eno3 | 0.0006342458119545198 |
| 90 | Tinagl1 | 0.0006320868660156235 |
| 1655 | Adap2 | 0.0006301084395605354 |
| 586 | Tspan8 | 0.0006244223824502777 |
| 1853 | St8sia4 | 0.0006240820727759114 |
| 1959 | Plxnd1 | 0.0006231885022156411 |
| 2909 | RGD1306271 | 0.0006217691538331295 |
| 2856 | AABR07030791.1 | 0.0006199015679973971 |
| 2672 | Zc3h12c | 0.0006168449709767086 |
| 363 | Upp1 | 0.0006056476951758856 |
| 2322 | Ripor2 | 0.0005819517766138432 |
| 1181 | AABR07038983.1 | 0.0005716051197370636 |
| 746 | Dapk2 | 0.0005713664560238957 |
| 313 | Lgals5 | 0.0005679411619606116 |
| 2314 | Alas1 | 0.0005641648633909341 |
| 1980 | Gimap8 | 0.0005639518113690252 |
| 2520 | Ttc9 | 0.0005590734497433977 |
| 3239 | ENSRNOG00000068881 | 0.0005490046803305838 |
| 3118 | Magi1 | 0.0005467837848080108 |
| 47 | Stmn1 | 0.0005425372007516173 |
| 2555 | St3gal6 | 0.0005405137171837497 |
| 471 | Lst1 | 0.0005384270308933447 |
| 924 | Sit1 | 0.0005348426069592574 |
| 1827 | Ptafr | 0.0005338890401116129 |
| 3108 | Smap2 | 0.0005328559006485239 |
| 112 | Nos2 | 0.0005327368846533384 |
| 1154 | Atp1a2 | 0.0005314758035442476 |
| 109 | Gzmk | 0.00053069175927129 |
| 616 | ENSRNOG00000060879 | 0.0005292282900376585 |
| 2028 | Shtn1 | 0.0005248838272524509 |
| 2257 | Fsip2 | 0.000521660644066693 |
| 2602 | Tec | 0.0005205438950382224 |
| 529 | Plet1 | 0.0005181565944573311 |
| 1319 | Tbc1d10c | 0.0005155617703388104 |
| 1272 | ENSRNOG00000066588 | 0.0005090902611212924 |
| 1157 | Hmgcs1 | 0.0005081898960587836 |
| 1573 | Ccm2l | 0.0005038391308759516 |
| 2579 | Krt23 | 0.0005019755386548884 |
| 995 | ENSRNOG00000003622 | 0.0005004454388636537 |
| 1789 | Foxf1 | 0.000496413930392171 |
| 2302 | Rapgef3 | 0.0004951536088193734 |
| 1014 | Lime1 | 0.0004939528248617496 |
| 1012 | Duoxa2 | 0.0004934929916204096 |
| 1100 | Dcn | 0.0004879489239731223 |
| 256 | Lmod1 | 0.00048656677433137334 |
| 2485 | Malt1 | 0.00048164016618953065 |
| 909 | Ereg | 0.0004812989471490202 |
| 2250 | Cenpu | 0.00047033384296642515 |
| 1851 | Cnr1 | 0.00046857423892084473 |
| 1962 | Cenpq | 0.0004647782108240451 |
| 3183 | Eomes | 0.00046468726119507146 |
| 2358 | Mybl2 | 0.0004614216722770416 |
| 25 | ENSRNOG00000068561 | 0.0004589389088548122 |
| 3018 | Gtse1 | 0.0004576572917580295 |
| 201 | Cd7 | 0.0004574227858424165 |
| 1636 | Cdca7l | 0.00045656023562290374 |
| 1997 | Ttk | 0.0004562571716341194 |
| 1848 | Epha2 | 0.0004540706282946292 |
| 1561 | AABR07039446.2 | 0.00045170610960918795 |
| 3222 | ENSRNOG00000063914 | 0.00044996676349089203 |
| 2215 | Foxred2 | 0.00044956106793798544 |
| 2914 | Ccdc146 | 0.00044931031412250017 |
| 2922 | Ndnf | 0.00044855173509288223 |
| 222 | RT1-Db1 | 0.00044278223215688233 |
| 1046 | Knstrn | 0.0004377358468183905 |
| 1257 | Ggt1 | 0.0004367672430858554 |
| 1852 | Gcnt1 | 0.00043638544163190354 |
| 28 | Cilp | 0.00043594526980250594 |
| 75 | Ly6c | 0.0004349604755340114 |
| 1798 | Nmu | 0.00043439358471191575 |
| 1581 | ENSRNOG00000049537 | 0.0004339624660557303 |
| 2597 | ENSRNOG00000065753 | 0.0004327295153248676 |
| 1908 | Matn2 | 0.0004315122002555048 |
| 1562 | ENSRNOG00000001027 | 0.00043052073919128044 |
| 2190 | B4galnt1 | 0.00042930481847660424 |
| 2240 | Il7 | 0.00042457036554345427 |
| 3084 | Sema4f | 0.00041831747865006715 |
| 1044 | Plbd1 | 0.0004179724893444316 |
| 1076 | Gp9 | 0.0004157606130743783 |
| 1666 | C3ar1 | 0.0004155683524048919 |
| 2573 | AABR07032097.1 | 0.00041469432796156303 |
| 737 | Glipr1 | 0.0004142734850617922 |
| 3105 | Lfng | 0.00041176910982081063 |
| 3209 | ENSRNOG00000066598 | 0.00041103178117835243 |
| 2645 | Spic | 0.00040999746032629417 |
| 1901 | Slc9a9 | 0.0004080418575886594 |
| 16 | Gzma | 0.00040757396905917345 |
| 2613 | Ikzf1 | 0.00040697305639966156 |
| 1865 | Rasgrp3 | 0.0004051868455074711 |
| 2664 | Upk1b | 0.0004049817893860629 |
| 977 | ENSRNOG00000033256 | 0.0004036439130321411 |
| 1894 | Etv1 | 0.0004019791316712617 |
| 636 | RGD1559482.1 | 0.0004018781819622196 |
| 2231 | Xkrx | 0.0003990081647237613 |
| 2822 | Wscd1 | 0.0003982117080788865 |
| 3079 | Cracd | 0.0003977409359781525 |
| 45 | Cdc45.1 | 0.0003936900009636049 |
| 2342 | Pik3cd | 0.00039274152922179947 |
| 1468 | Npnt | 0.0003921357554558285 |
| 1512 | Scnn1b | 0.0003921341309008492 |
| 2761 | Pik3cg | 0.00039200203184779764 |
| 447 | Cst7 | 0.0003893747456423356 |
| 2386 | Tal1 | 0.0003890937213426842 |
| 1883 | ENSRNOG00000032970 | 0.00038141892224882235 |
| 1051 | Lpar6 | 0.00038070171028051106 |
| 54 | Des | 0.0003796509138234393 |
| 2907 | ENSRNOG00000070888 | 0.0003782577744677116 |
| 1024 | Nuf2 | 0.000378110952269102 |
| 1880 | Adssl1 | 0.0003762923917788279 |
| 158 | Cd8a | 0.0003756430819760534 |
| 325 | Fam110d | 0.0003754943640185888 |
| 345 | Il4i1 | 0.000374156283596238 |
| 2327 | Egfl8 | 0.000374065144299661 |
| 327 | Spry1 | 0.000373158457307624 |
| 984 | Heph | 0.00037254616759975354 |
| 3207 | Slc16a7 | 0.00037095609752231377 |
| 3130 | Slc7a2 | 0.00036947110487275134 |
| 2109 | Ptpn22 | 0.0003688896757963229 |
| 958 | Pacsin3 | 0.0003678996989077142 |
| 2168 | Nrp2 | 0.0003637912613534981 |
| 1384 | Itk | 0.00036310088209390025 |
| 1746 | Cd37 | 0.0003611075357640803 |
| 2347 | P2rx1 | 0.0003596399474608123 |
| 1474 | Tnfrsf14 | 0.0003591737312464417 |
| 1786 | Slit2 | 0.0003584152090209533 |
| 379 | Pdgfa | 0.0003578040178073219 |
| 647 | Chp2 | 0.0003520687870496244 |
| 3001 | Ppp1r14a | 0.0003483911030915182 |
| 1946 | Cenpt | 0.00034679879224769057 |
| 598 | Cxcr6 | 0.00034493124241470103 |
| 3215 | Aknad1 | 0.0003422450909955954 |
| 3083 | Iqgap3 | 0.00034197326978778887 |
| 1526 | Il7r | 0.00034188804891548445 |
| 587 | Tnfrsf4 | 0.00034060781676631283 |
| 1156 | Lilrb4 | 0.00033938516714864284 |
| 642 | ENSRNOG00000061714 | 0.000337909478260879 |
| 1719 | Rad51ap2 | 0.00033503740719273325 |
| 908 | Esco2 | 0.0003313150774910292 |
| 2742 | ENSRNOG00000071065 | 0.0003274907180848449 |
| 502 | Kif11 | 0.00032528063609589563 |
| 1304 | Itgax | 0.0003235435391005107 |
| 474 | Lgalsl | 0.0003230537091573739 |
| 2773 | Scarf2 | 0.0003185031405577831 |
| 2666 | Fgf1 | 0.0003172024248198794 |
| 2172 | Inf2 | 0.0003133201066872427 |
| 1642 | Slc15a2 | 0.00031247933787758317 |
| 1760 | Pou2f2 | 0.0003113581744490361 |
| 1714 | Rab11fip1 | 0.00031069390416796426 |
| 275 | ENSRNOG00000068089 | 0.00030955426102519714 |
| 495 | Cadm1 | 0.0003049372124426141 |
| 655 | Foxs1 | 0.00029888409681060973 |
| 3050 | ENSRNOG00000070250 | 0.0002975077539156009 |
| 1321 | Txk | 0.00029680438738083554 |
| 409 | Clec9a | 0.0002953957979535815 |
| 2417 | Ctnnal1 | 0.0002946492856676804 |
| 174 | Ccl22 | 0.0002937399451514638 |
| 1395 | Ms4a6bl | 0.00029283730503460494 |
| 858 | Itga6 | 0.0002898750659520686 |
| 1747 | Vstm1 | 0.00028923762571984174 |
| 432 | Tgm2 | 0.00028584658932243046 |
| 2675 | ENSRNOG00000007682 | 0.0002847074349834475 |
| 2236 | Kcnn4 | 0.00028317152952863863 |
| 1932 | ENSRNOG00000066887 | 0.00028286509466887277 |
| 896 | Klf5 | 0.00028231836223402103 |
| 557 | Klri1 | 0.0002819482500551348 |
| 515 | Cd3d | 0.00028135971605911653 |
| 2000 | Pde1a | 0.00028107700813269115 |
| 646 | Ly49s6 | 0.0002799556256728664 |
| 182 | Cav1 | 0.00027913650172024096 |
| 854 | ENSRNOG00000063179 | 0.00027782267493285087 |
| 2007 | St3gal5 | 0.0002778115888801332 |
| 198 | Rem1 | 0.0002756574426697921 |
| 2046 | Kcnk1 | 0.00027556761276721376 |
| 1737 | Stap1 | 0.00027508675297222354 |
| 1861 | Camk4 | 0.00027413848491988804 |
| 63 | Tpsab1 | 0.000272267715964365 |
| 274 | Prok2 | 0.0002691564480877995 |
| 1432 | Gimap1 | 0.00026788787830183845 |
| 2363 | Inpp4b | 0.0002651362978557633 |
| 24 | ENSRNOG00000070540 | 0.0002632061841453104 |
| 461 | Rasip1 | 0.0002625472632358421 |
| 754 | Amigo2 | 0.00025979745384376973 |
| 1139 | Olr1585 | 0.0002593397585327144 |
| 2699 | ENSRNOG00000064228 | 0.0002566264439703843 |
| 2488 | ENSRNOG00000064540 | 0.00025502837847466456 |
| 2502 | Stox1 | 0.00025287541826080745 |
| 1342 | Fgd5 | 0.0002520866687997993 |
| 438 | Anxa8 | 0.0002500478556990891 |
| 2910 | Mtmr10 | 0.0002497671526008198 |
| 2429 | Htra4 | 0.00024313942510216876 |
| 1308 | Itgb2 | 0.00024298558901438352 |
| 2529 | Lilrb2 | 0.000242157767593609 |
| 2330 | Oma1 | 0.00024190668457507454 |
| 181 | Lrg1 | 0.00024111761382105025 |
| 2146 | Nwd1 | 0.00023642781437249936 |
| 3131 | Alk | 0.000235999990348154 |
| 1555 | Stac | 0.00023522078912810622 |
| 1424 | Stc1 | 0.00023490838685723107 |
| 2826 | Tchh.1 | 0.00023403135026775149 |
| 873 | Upk3bl1 | 0.0002333265714090868 |
| 2680 | Gjc2 | 0.00023323136932665994 |
| 3231 | ENSRNOG00000064824 | 0.00022853127200999637 |
| 2313 | Etv4 | 0.00022847920308933294 |
| 2697 | Etl4 | 0.0002266001897020392 |
| 3008 | Dock6 | 0.00022619184117187786 |
| 1689 | Fgr | 0.00022527760687077127 |
| 2776 | Snai3 | 0.00022496453150037775 |
| 1585 | Fam83d | 0.00022392469775632296 |
| 1622 | ENSRNOG00000070434 | 0.00022370825047247195 |
| 1303 | Zcchc12 | 0.000223046335385761 |
| 2731 | ENSRNOG00000070558 | 0.00022093084053242235 |
| 1854 | Oscar | 0.00022053042765701752 |
| 147 | Dusp2 | 0.00021992735054286624 |
| 717 | Fcrl5 | 0.00021802483777613637 |
| 767 | Ano1 | 0.00021686137845261047 |
| 3062 | Bco1 | 0.00021682493299769948 |
| 2463 | Prr15 | 0.00021254963402552422 |
| 86 | Egr4 | 0.00021183116293003822 |
| 2847 | Serpinb8 | 0.00021076995114534692 |
| 3080 | ENSRNOG00000067004 | 0.00020916723684983912 |
| 2074 | Gcnt2 | 0.00020564220747555954 |
| 2278 | Ciita | 0.00020541208116086956 |
| 2189 | ENSRNOG00000037845 | 0.00020527379709030436 |
| 1886 | Adamts8 | 0.0002049466677135816 |
| 1064 | Pld4 | 0.00019939641830236135 |
| 190 | Fcrla | 0.0001984869154050534 |
| 2604 | Pgam2 | 0.0001966195599479108 |
| 2048 | ENSRNOG00000066647 | 0.00019618228105550744 |
| 1125 | Neb | 0.00019427562163391052 |
| 2546 | Prima1 | 0.00019400761078229486 |
| 2875 | Ccne2 | 0.00019274758293163523 |
| 1088 | Ikzf3 | 0.00019170243198368626 |
| 2685 | Fam13c | 0.00019141002269615028 |
| 718 | Myl1 | 0.0001887372019781129 |
| 2894 | Nrxn2 | 0.00018657532136309187 |
| 364 | Xcr1 | 0.00018485026957928253 |
| 2863 | Ddit4l | 0.00018354205289407023 |
| 1735 | Nrap | 0.00018282181870337032 |
| 3100 | Ggnbp1 | 0.00018149170065998653 |
| 1717 | Lbh | 0.0001803485135014962 |
| 2575 | Auts2l1 | 0.0001801395917861888 |
| 3065 | AABR07030771.1 | 0.00017649145937381435 |
| 1549 | Zap70 | 0.00017457971116368947 |
| 1993 | Ttc6 | 0.0001745579894747025 |
| 2793 | Anln | 0.00017425036065663984 |
| 1010 | ENSRNOG00000011702 | 0.0001727431077930728 |
| 1628 | NEWGENE-2134 | 0.00016369822483966727 |
| 1715 | Snx20 | 0.0001636223018801353 |
| 284 | Mmrn2 | 0.00015626319853024115 |
| 2174 | Fbxl22 | 0.00015600202718170085 |
| 223 | ENSRNOG00000067090 | 0.0001544525301249516 |
| 1008 | Nlrp3 | 0.00015338730848815673 |
| 416 | ENSRNOG00000065655 | 0.0001516036941319528 |
| 445 | Tesc | 0.00015041171901502014 |
| 1213 | Mcm5 | 0.00015017898466555547 |
| 531 | Acta1 | 0.0001496986850240302 |
| 2614 | Parvb | 0.00014880752524743782 |
| 5 | ENSRNOG00000070861 | 0.0001472290469137658 |
| 383 | Gja4 | 0.00014694834255397595 |
| 1836 | Inka2 | 0.0001469460050592485 |
| 2879 | Pkmyt1 | 0.00014480409760717606 |
| 842 | Il1rn | 0.0001439105816268779 |
| 2297 | Capn6 | 0.0001423461321368433 |
| 1258 | Rprm | 0.0001408453073658559 |
| 2178 | Krt75 | 0.00013898333476474156 |
| 2445 | Sytl3 | 0.00013694322255826944 |
| 2405 | Gng8 | 0.00013596588557200564 |
| 574 | Pla2g7 | 0.0001332310951787517 |
| 1054 | Adh6 | 0.000128515448895324 |
| 169 | Ndufa4l2 | 0.00012703456710133404 |
| 560 | ENSRNOG00000068062 | 0.00012620297920536502 |
| 1876 | Tp53i11 | 0.0001258766178423078 |
| 1426 | Cndp1 | 0.00012491270586485964 |
| 2882 | Hfe | 0.00012030222505788902 |
| 202 | Klrb1a | 0.0001185153760128782 |
| 473 | Tacr1 | 0.00011836368869085458 |
| 314 | Cd163 | 0.00011736537662151297 |
| 950 | Spn | 0.00011705975214031266 |
| 1412 | Asb2 | 0.00011464059145302175 |
| 1578 | Ncr3 | 0.00011339281905359312 |
| 808 | Prox1 | 0.00011123027064537717 |
| 428 | Olr1 | 0.00011093592153622685 |
| 78 | Top2a | 0.00010951772765518082 |
| 3206 | Camk2b | 0.00010931352752066509 |
| 1450 | Sorl1 | 0.00010875884670571419 |
| 1172 | Cdca8 | 0.0001067467086139715 |
| 2473 | Chn1 | 0.00010466320412657408 |
| 1397 | Gpr4 | 0.00010398407198958263 |
| 1079 | Bcl11a | 0.0001004282967805543 |
| 2472 | Gria4 | 9.48134973825524e-05 |
| 1999 | Tlr11 | 9.11977932099641e-05 |
| 2801 | ENSRNOG00000064277 | 9.089649468437385e-05 |
| 2585 | Fmo5 | 8.818462562282372e-05 |
| 405 | Nexn | 8.759124942734597e-05 |
| 592 | Gata2 | 8.64307417237839e-05 |
| 1779 | B3gnt5 | 8.415900238179485e-05 |
| 1921 | Cobll1 | 8.386025700607972e-05 |
| 1021 | Map4k1 | 7.994769700360639e-05 |
| 1090 | Ajuba | 7.981178023673966e-05 |
| 2975 | Rgs18 | 7.907157929823485e-05 |
| 1387 | Dsp | 7.787420957038467e-05 |
| 690 | Tnfsf11 | 7.559900099370304e-05 |
| 1541 | Bcar3 | 7.478952001973534e-05 |
| 254 | Sox17 | 7.336967897719663e-05 |
| 703 | Slc13a3 | 7.278158886945603e-05 |
| 176 | RGD1310587 | 7.193631807316837e-05 |
| 2434 | Dock8 | 7.058304124022993e-05 |
| 2272 | ENSRNOG00000071212 | 6.824963245700806e-05 |
| 2618 | Notch1 | 6.67036012268748e-05 |
| 1667 | ENSRNOG00000068731 | 6.651536578859929e-05 |
| 67 | Ifitm6 | 6.401892532177756e-05 |
| 1739 | ENSRNOG00000069051 | 6.224559397222324e-05 |
| 303 | Shisa3 | 6.186273163991093e-05 |
| 1425 | Gpr132 | 6.12055635874528e-05 |
| 2981 | Fbn2 | 5.959170435990171e-05 |
| 537 | Cxcr2 | 5.828757851591961e-05 |
| 84 | Ccl21 | 5.8275853659093575e-05 |
| 771 | Cst6 | 5.482784441103011e-05 |
| 2179 | Ica1 | 5.480685741441796e-05 |
| 1162 | Clspn | 5.29496967213696e-05 |
| 615 | Runx3 | 5.196545944758139e-05 |
| 1566 | Tff3 | 4.701118806541874e-05 |
| 68 | Ccl12 | 4.700270421190084e-05 |
| 1041 | Fgf9 | 4.6082367366304664e-05 |
| 770 | ENSRNOG00000062480 | 4.375578248003933e-05 |
| 1089 | RT1-DOa | 4.074322705769032e-05 |
| 2772 | ENSRNOG00000031058 | 3.9792594772465384e-05 |
| 829 | Il1a | 3.963350000763496e-05 |
| 1065 | Pou2af1 | 3.69389071847403e-05 |
| 180 | Rgs4 | 3.4639385535872776e-05 |
| 3102 | Slc37a2 | 3.402480483461047e-05 |
| 2789 | She | 3.327232629876737e-05 |
| 494 | Klrc3 | 3.1114252102968994e-05 |
| 627 | Msln | 2.9611701690225792e-05 |
| 106 | Stfa2l2 | 2.8367351626993752e-05 |
| 2101 | Rassf10 | 2.5207492540086754e-05 |
| 1232 | Pkib | 2.268483241983768e-05 |
| 853 | Sgo2 | 2.2064908445332124e-05 |
| 661 | Clec7a | 2.1767608591046126e-05 |
| 2130 | Ly49s7 | 2.041980187737967e-05 |
| 1808 | Slc11a1 | 2.0330221626382336e-05 |
| 2933 | Oip5 | 1.9974113563488988e-05 |
| 2214 | Tnnc2 | 1.965139866214576e-05 |
| 213 | Tmem252 | 1.9381246615033763e-05 |
| 73 | ENSRNOG00000069624 | 1.7183442543173966e-05 |
| 571 | Gcsam | 1.5059235751661895e-05 |
| 2817 | Slpil2.1 | 7.83493162117311e-06 |
| 2601 | Ly49si1 | 6.835652671334161e-06 |
| 2055 | Cldn8 | 6.44790215324884e-06 |
| 1632 | ENSRNOG00000069947 | 3.7673079157513406e-06 |
| 1925 | Atp9a | 3.1449924673201457e-06 |
| 3137 | Tnfsf14 | 2.6419248539340735e-06 |
| 104 | Ccr7 | 1.1400551915541244e-06 |
| 1532 | Fetub | 3.4130135232032806e-07 |
| 2333 | ENSRNOG00000032729 | 0.0 |
| 2334 | Gpr35 | 0.0 |
| 743 | ENSRNOG00000063173 | 0.0 |
| 2295 | Arhgef39 | 0.0 |
| 2300 | Lcn8 | 0.0 |
| 2307 | Thsd7a | 0.0 |
| 1164 | Foxa1 | 0.0 |
| 1169 | Tnnt2 | 0.0 |
| 747 | Uhrf1 | 0.0 |
| 2316 | ENSRNOG00000049289 | 0.0 |
| 2319 | Kcnc3 | 0.0 |
| 2320 | Lpal2 | 0.0 |
| 759 | Nrip2 | 0.0 |
| 939 | Cd27 | 0.0 |
| 942 | Klri2 | 0.0 |
| 1098 | Klk6 | 0.0 |
| 1099 | Cyp26a1 | 0.0 |
| 1106 | Cyp2e1 | 0.0 |
| 2004 | AABR07027866.1 | 0.0 |
| 2010 | ENSRNOG00000068518 | 0.0 |
| 2014 | Lmod2 | 0.0 |
| 959 | Olfm4 | 0.0 |
| 2355 | Tmprss4 | 0.0 |
| 726 | Asf1b | 0.0 |
| 727 | Aplnr | 0.0 |
| 733 | Tbx1 | 0.0 |
| 735 | ENSRNOG00000062856 | 0.0 |
| 739 | Trh | 0.0 |
| 745 | Col28a1 | 0.0 |
| 1950 | Snap25 | 0.0 |
| 1951 | Pear1 | 0.0 |
| 1952 | Trem3 | 0.0 |
| 1955 | Mmp7 | 0.0 |
| 1158 | ENSRNOG00000068207 | 0.0 |
| 1159 | Skap1 | 0.0 |
| 1160 | Kif18b | 0.0 |
| 1163 | Perp | 0.0 |
| 2371 | Dcstamp | 0.0 |
| 710 | Pawr | 0.0 |
| 1924 | Cyfip2 | 0.0 |
| 1926 | Scgb3a1 | 0.0 |
| 1928 | Tspan33 | 0.0 |
| 1933 | S1pr5 | 0.0 |
| 1937 | Kif14 | 0.0 |
| 1938 | Sbspon | 0.0 |
| 1939 | Pdzk1 | 0.0 |
| 1940 | Adam12 | 0.0 |
| 751 | Espn | 0.0 |
| 2341 | Myo10 | 0.0 |
| 2343 | ENSRNOG00000062779 | 0.0 |
| 2346 | Dclk3 | 0.0 |
| 2352 | Fhl5 | 0.0 |
| 1949 | Adamtsl2 | 0.0 |
| 716 | Ahsp | 0.0 |
| 2374 | Adrb1 | 0.0 |
| 2375 | Sytl1 | 0.0 |
| 2381 | Spock3 | 0.0 |
| 696 | Mill1 | 0.0 |
| 697 | Mmp9 | 0.0 |
| 701 | Tspan12 | 0.0 |
| 702 | Kif20a | 0.0 |
| 2400 | ENSRNOG00000064524 | 0.0 |
| 2362 | Timd2 | 0.0 |
| 2364 | Mlip | 0.0 |
| 2366 | Art4 | 0.0 |
| 678 | Mrgprb3 | 0.0 |
| 679 | ENSRNOG00000026336 | 0.0 |
| 680 | Cd5l | 0.0 |
| 681 | ENSRNOG00000066355 | 0.0 |
| 683 | Reln | 0.0 |
| 685 | Tlr12 | 0.0 |
| 1912 | ENSRNOG00000071120 | 0.0 |
| 1913 | Olr63 | 0.0 |
| 1914 | Ncapg | 0.0 |
| 1920 | ENSRNOG00000067151 | 0.0 |
| 1173 | Nkx3-1 | 0.0 |
| 1175 | Prnd | 0.0 |
| 1177 | Bdnf | 0.0 |
| 1941 | Nek2l1 | 0.0 |
| 1203 | Mall | 0.0 |
| 1899 | ENSRNOG00000071162 | 0.0 |
| 1178 | Faim2 | 0.0 |
| 1186 | Scart1 | 0.0 |
| 689 | Pdcd1 | 0.0 |
| 2415 | Rab3b | 0.0 |
| 2419 | Irx4 | 0.0 |
| 2420 | Zfp488 | 0.0 |
| 2421 | Klrh1 | 0.0 |
| 2422 | ENSRNOG00000063334 | 0.0 |
| 2424 | ENSRNOG00000069245 | 0.0 |
| 2427 | Apcdd1l | 0.0 |
| 2430 | Arhgef15 | 0.0 |
| 677 | Mmp13 | 0.0 |
| 651 | Siglec10 | 0.0 |
| 653 | Sctr | 0.0 |
| 654 | Il13ra2 | 0.0 |
| 656 | ENSRNOG00000000053 | 0.0 |
| 658 | Tcap | 0.0 |
| 2431 | E2f8 | 0.0 |
| 2435 | Gins1 | 0.0 |
| 660 | Ccdc184 | 0.0 |
| 663 | Ppp1r1c | 0.0 |
| 665 | Chmp4c | 0.0 |
| 667 | Col11a1 | 0.0 |
| 671 | Tspan15 | 0.0 |
| 1193 | Trhr | 0.0 |
| 1195 | ENSRNOG00000070500 | 0.0 |
| 635 | Cilp2 | 0.0 |
| 638 | Gimap9 | 0.0 |
| 640 | Gucy1b1 | 0.0 |
| 641 | Ostn | 0.0 |
| 2474 | Dmp1 | 0.0 |
| 2482 | Edil3 | 0.0 |
| 1909 | Plpp4 | 0.0 |
| 1910 | Hhip | 0.0 |
| 675 | Gja5 | 0.0 |
| 2437 | Stra6 | 0.0 |
| 2442 | Gfi1 | 0.0 |
| 2444 | Cmtm8 | 0.0 |
| 2451 | Trim63 | 0.0 |
| 2466 | Defb52 | 0.0 |
| 2155 | Fzd10 | 0.0 |
| 2156 | Kcnj10 | 0.0 |
| 2157 | Tp63 | 0.0 |
| 2158 | Grm4 | 0.0 |
| 2159 | Sema3f | 0.0 |
| 2162 | Zic2 | 0.0 |
| 2166 | Map2 | 0.0 |
| 2167 | Prlr | 0.0 |
| 2169 | Fam71e2 | 0.0 |
| 2171 | ENSRNOG00000064255 | 0.0 |
| 954 | Chad | 0.0 |
| 957 | ENSRNOG00000070677 | 0.0 |
| 1081 | Clec1b | 0.0 |
| 634 | Adipoq | 0.0 |
| 2217 | RGD1563354 | 0.0 |
| 2218 | Krt79 | 0.0 |
| 2224 | Grifin | 0.0 |
| 2226 | ENSRNOG00000062454 | 0.0 |
| 2227 | Ascl1 | 0.0 |
| 869 | AABR07021213.1 | 0.0 |
| 870 | Meox1 | 0.0 |
| 872 | Mecom | 0.0 |
| 1888 | ENSRNOG00000069233 | 0.0 |
| 1890 | Cd19 | 0.0 |
| 1188 | Vgf | 0.0 |
| 624 | Ly49i2 | 0.0 |
| 659 | Mt3 | 0.0 |
| 2455 | Msr1 | 0.0 |
| 2460 | Ky | 0.0 |
| 1872 | ENSRNOG00000004133 | 0.0 |
| 618 | Wnt10a | 0.0 |
| 620 | RGD1559482 | 0.0 |
| 1215 | Gzmbl1 | 0.0 |
| 1219 | Ms4a4c | 0.0 |
| 1878 | Bank1 | 0.0 |
| 2003 | Ccn6 | 0.0 |
| 1109 | ENSRNOG00000063720 | 0.0 |
| 1117 | ENSRNOG00000067275 | 0.0 |
| 1118 | Cxcr3 | 0.0 |
| 1120 | Fbln7 | 0.0 |
| 2504 | Myh14 | 0.0 |
| 2505 | Akr7a3 | 0.0 |
| 2507 | Cnga4 | 0.0 |
| 2508 | Nrg1 | 0.0 |
| 581 | Crlf1 | 0.0 |
| 588 | Kif20b | 0.0 |
| 2490 | Wnt5b | 0.0 |
| 2497 | Tmem150c | 0.0 |
| 596 | Ly49i3 | 0.0 |
| 605 | Heyl | 0.0 |
| 606 | Sox9 | 0.0 |
| 1230 | Sgip1 | 0.0 |
| 1863 | Myct1 | 0.0 |
| 1870 | Car7 | 0.0 |
| 568 | Serpinb10 | 0.0 |
| 572 | Klrg1 | 0.0 |
| 2537 | Nmur1 | 0.0 |
| 2540 | Mfng | 0.0 |
| 2541 | Lypd1 | 0.0 |
| 1874 | ENSRNOG00000067817 | 0.0 |
| 1205 | Melk | 0.0 |
| 1208 | Cep55 | 0.0 |
| 608 | ENSRNOG00000065206 | 0.0 |
| 611 | Tnip3 | 0.0 |
| 2500 | RGD1308065 | 0.0 |
| 2501 | Kcnma1 | 0.0 |
| 2503 | Fut9 | 0.0 |
| 558 | Slc22a3 | 0.0 |
| 561 | Ly49i5 | 0.0 |
| 1845 | Gcgr | 0.0 |
| 1850 | Cfap73 | 0.0 |
| 1859 | Foxd1 | 0.0 |
| 1220 | Ckm | 0.0 |
| 1221 | Scn3a | 0.0 |
| 1223 | Cldn11 | 0.0 |
| 594 | Irf8 | 0.0 |
| 595 | AABR07030773.1 | 0.0 |
| 2526 | ENSRNOG00000063458 | 0.0 |
| 2530 | Spag5 | 0.0 |
| 2531 | Tnfrsf11a | 0.0 |
| 1229 | Fam107a | 0.0 |
| 563 | Gimap7 | 0.0 |
| 2556 | Capn13 | 0.0 |
| 2557 | Fhod3 | 0.0 |
| 2560 | ENSRNOG00000070219 | 0.0 |
| 2562 | Cbln1 | 0.0 |
| 2564 | Il17rb | 0.0 |
| 2566 | Mkx | 0.0 |
| 2567 | ENSRNOG00000067596 | 0.0 |
| 2568 | ENSRNOG00000066423 | 0.0 |
| 2569 | Cttnbp2 | 0.0 |
| 2570 | Clec2d2 | 0.0 |
| 2571 | Ckap2l | 0.0 |
| 2574 | Rnf144b | 0.0 |
| 552 | Gimap4 | 0.0 |
| 556 | Cx3cr1 | 0.0 |
| 987 | Ceacam4 | 0.0 |
| 534 | Cacng3 | 0.0 |
| 535 | Pgm5 | 0.0 |
| 536 | Pdlim3 | 0.0 |
| 1840 | Lypd8 | 0.0 |
| 1841 | ENSRNOG00000070324 | 0.0 |
| 1842 | Kcna1 | 0.0 |
| 1843 | Dipk1c | 0.0 |
| 1236 | AABR07017733.1 | 0.0 |
| 1237 | Cbs | 0.0 |
| 1238 | Cdkn2a | 0.0 |
| 1242 | Scnn1g | 0.0 |
| 2598 | Gpr84 | 0.0 |
| 2599 | Mycn | 0.0 |
| 2576 | MGC109340.1 | 0.0 |
| 2577 | ENSRNOG00000066174 | 0.0 |
| 2578 | Wwc1 | 0.0 |
| 1082 | Fcer2 | 0.0 |
| 1084 | ENSRNOG00000065346 | 0.0 |
| 1087 | Pax1 | 0.0 |
| 1060 | Cd28 | 0.0 |
| 1062 | Alox15 | 0.0 |
| 1826 | Slco4a1 | 0.0 |
| 1829 | Abcb1b.1 | 0.0 |
| 1831 | Nfe2 | 0.0 |
| 1834 | Cx3cl1 | 0.0 |
| 1837 | Hspb3 | 0.0 |
| 1243 | Tnfrsf17 | 0.0 |
| 1249 | Bmp5 | 0.0 |
| 1251 | Spc25 | 0.0 |
| 545 | Irag1 | 0.0 |
| 2580 | Zp1 | 0.0 |
| 2582 | Gria1 | 0.0 |
| 2587 | Slc30a2 | 0.0 |
| 2593 | ENSRNOG00000070399 | 0.0 |
| 2625 | Ankrd55 | 0.0 |
| 2626 | F5 | 0.0 |
| 503 | Cryaa | 0.0 |
| 505 | Lta | 0.0 |
| 511 | Nap1l5 | 0.0 |
| 512 | Dll4 | 0.0 |
| 513 | Tac3 | 0.0 |
| 2630 | AC109737.1 | 0.0 |
| 2631 | Xkr4 | 0.0 |
| 2609 | Slc7a10 | 0.0 |
| 516 | Ltbp2 | 0.0 |
| 520 | Ca4 | 0.0 |
| 527 | Npy | 0.0 |
| 1267 | Smoc1 | 0.0 |
| 1820 | Rnf182 | 0.0 |
| 2638 | Syt13 | 0.0 |
| 2642 | Nxpe5 | 0.0 |
| 484 | Sostdc1 | 0.0 |
| 490 | Gapt | 0.0 |
| 497 | Chst13 | 0.0 |
| 1280 | Cacng6 | 0.0 |
| 1261 | RGD1561143 | 0.0 |
| 1266 | Pkhd1l1 | 0.0 |
| 1839 | Spink5 | 0.0 |
| 2619 | Arpp21 | 0.0 |
| 2621 | AABR07026557.1 | 0.0 |
| 1273 | Zdhhc2 | 0.0 |
| 2646 | Tnfsf8 | 0.0 |
| 2647 | Clec4e | 0.0 |
| 2648 | Col8a2 | 0.0 |
| 2653 | C10H5orf58 | 0.0 |
| 2654 | Cym | 0.0 |
| 2657 | Gpr182 | 0.0 |
| 2658 | Cdca7 | 0.0 |
| 476 | Agt | 0.0 |
| 478 | Ms4a1 | 0.0 |
| 482 | Klra1 | 0.0 |
| 483 | Ccr9 | 0.0 |
| 2660 | Tm6sf2 | 0.0 |
| 2632 | Muc20 | 0.0 |
| 2634 | Robo3 | 0.0 |
| 439 | Spib | 0.0 |
| 443 | Gal | 0.0 |
| 444 | Itih3 | 0.0 |
| 449 | Clec1a | 0.0 |
| 2692 | Trim55 | 0.0 |
| 2696 | Sema6b | 0.0 |
| 2668 | Phf11b | 0.0 |
| 2670 | Eva1a | 0.0 |
| 455 | Kcna5 | 0.0 |
| 456 | Ncam1 | 0.0 |
| 1288 | ENSRNOG00000053656 | 0.0 |
| 1782 | Tmem132c | 0.0 |
| 1791 | Ppp1r14c | 0.0 |
| 1793 | Gprin3 | 0.0 |
| 2201 | ENSRNOG00000064012 | 0.0 |
| 2210 | Hist1h2bc | 0.0 |
| 2211 | ENSRNOG00000064081 | 0.0 |
| 884 | ENSRNOG00000068082 | 0.0 |
| 885 | Aloxe3 | 0.0 |
| 888 | Parm1 | 0.0 |
| 891 | Kif23 | 0.0 |
| 895 | Ednra | 0.0 |
| 1772 | Paqr5 | 0.0 |
| 1773 | Slc26a10 | 0.0 |
| 467 | Ctxn3 | 0.0 |
| 2681 | Cebpe | 0.0 |
| 2683 | ENSRNOG00000016480 | 0.0 |
| 2691 | Slurp2 | 0.0 |
| 2704 | Chst7 | 0.0 |
| 421 | Xirp1 | 0.0 |
| 422 | Ccna2 | 0.0 |
| 424 | Ntrk1 | 0.0 |
| 1307 | Ushbp1 | 0.0 |
| 1309 | Ankrd65 | 0.0 |
| 1312 | Clic5 | 0.0 |
| 1315 | Prph | 0.0 |
| 1770 | Apobec2 | 0.0 |
| 874 | Lamc3 | 0.0 |
| 876 | Slc2a4 | 0.0 |
| 2229 | ENSRNOG00000070827 | 0.0 |
| 2232 | Tacstd2 | 0.0 |
| 915 | Vpreb3 | 0.0 |
| 2199 | Hjurp | 0.0 |
| 2200 | Tgm3 | 0.0 |
| 1285 | Podnl1 | 0.0 |
| 1286 | AC097037.1 | 0.0 |
| 1538 | Wfikkn2 | 0.0 |
| 430 | Ccl19 | 0.0 |
| 431 | Cnn1 | 0.0 |
| 2713 | Il23r | 0.0 |
| 2717 | Mtmr7 | 0.0 |
| 2719 | ENSRNOG00000008450 | 0.0 |
| 2723 | Gpr18 | 0.0 |
| 2729 | Zcchc18 | 0.0 |
| 3220 | ENSRNOG00000057332 | 0.0 |
| 2735 | Crybg1 | 0.0 |
| 2736 | Chrm2 | 0.0 |
| 2738 | Wnt6 | 0.0 |
| 404 | Cxcl11 | 0.0 |
| 2703 | Inhba | 0.0 |
| 2139 | ENSRNOG00000070418 | 0.0 |
| 82 | Depp1 | 0.0 |
| 3205 | St8sia6 | 0.0 |
| 1011 | Tbx2 | 0.0 |
| 1519 | Tnfrsf13c | 0.0 |
| 2104 | Kcns2 | 0.0 |
| 1509 | Gja3 | 0.0 |
| 419 | Mcam | 0.0 |
| 2741 | Tfec | 0.0 |
| 1326 | Ocstamp | 0.0 |
| 1328 | ENSRNOG00000070606 | 0.0 |
| 1330 | Gldn | 0.0 |
| 1748 | Sapcd2 | 0.0 |
| 1753 | ENSRNOG00000050006 | 0.0 |
| 1754 | Pcp4 | 0.0 |
| 1301 | Car2 | 0.0 |
| 1738 | Pllp | 0.0 |
| 3233 | Tfpi2 | 0.0 |
| 1744 | Tox | 0.0 |
| 3188 | Enpp6 | 0.0 |
| 407 | Cd300lg | 0.0 |
| 412 | Cxcl14 | 0.0 |
| 413 | Cd8b | 0.0 |
| 2117 | Pde6h | 0.0 |
| 2125 | Gfra3 | 0.0 |
| 2128 | Adamts3 | 0.0 |
| 2129 | ENSRNOG00000063803 | 0.0 |
| 3185 | Sgo1 | 0.0 |
| 968 | Aspm | 0.0 |
| 972 | Cxcl6 | 0.0 |
| 979 | ENSRNOG00000049919 | 0.0 |
| 2136 | Slc7a11 | 0.0 |
| 2768 | Chst3 | 0.0 |
| 2769 | Pacrg | 0.0 |
| 2770 | Nlrp12 | 0.0 |
| 373 | Ifng | 0.0 |
| 3192 | Frzb | 0.0 |
| 387 | Camp | 0.0 |
| 17 | Xcl1 | 0.0 |
| 2743 | Ankrd66 | 0.0 |
| 2744 | Cass4 | 0.0 |
| 2745 | Hist1h3b | 0.0 |
| 2746 | Pappa1 | 0.0 |
| 2747 | Megf6 | 0.0 |
| 2752 | Scx | 0.0 |
| 388 | Egfl6 | 0.0 |
| 392 | Il12b | 0.0 |
| 395 | Angptl7 | 0.0 |
| 2777 | Slco1a4 | 0.0 |
| 2787 | Traip | 0.0 |
| 356 | Synpo2 | 0.0 |
| 19 | ENSRNOG00000062317 | 0.0 |
| 1722 | ENSRNOG00000066332 | 0.0 |
| 1727 | Gimap6 | 0.0 |
| 1731 | ENSRNOG00000064682 | 0.0 |
| 1733 | Kifc1 | 0.0 |
| 3210 | Cntnap2 | 0.0 |
| 3194 | Sh2b2 | 0.0 |
| 1316 | Dll1 | 0.0 |
| 1318 | Egflam | 0.0 |
| 1324 | ENSRNOG00000002858 | 0.0 |
| 2756 | Clic3 | 0.0 |
| 2757 | ENSRNOG00000063477 | 0.0 |
| 2758 | Lratd1 | 0.0 |
| 369 | Cd3g | 0.0 |
| 2788 | ENSRNOG00000068569 | 0.0 |
| 3197 | Arhgap26 | 0.0 |
| 2792 | P4ha3 | 0.0 |
| 3201 | Parpbp | 0.0 |
| 3202 | Ptpn5 | 0.0 |
| 2805 | Hk3 | 0.0 |
| 1359 | Hist3h2ba | 0.0 |
| 1360 | Bub1b | 0.0 |
| 1362 | ENSRNOG00000069285 | 0.0 |
| 1706 | Kcnk7 | 0.0 |
| 1707 | Cpne4 | 0.0 |
| 1711 | Itga8 | 0.0 |
| 20 | Gpihbp1 | 0.0 |
| 21 | Tpsb2 | 0.0 |
| 3179 | Shank2 | 0.0 |
| 1354 | ENSRNOG00000067544 | 0.0 |
| 339 | Sfrp5 | 0.0 |
| 1334 | Adgrg3 | 0.0 |
| 2807 | Pnmt | 0.0 |
| 2810 | Crtam | 0.0 |
| 2811 | F2rl2 | 0.0 |
| 2815 | Cdhr1 | 0.0 |
| 3180 | Prom1 | 0.0 |
| 344 | Podxl | 0.0 |
| 351 | Sorbs2 | 0.0 |
| 353 | Olr292.1 | 0.0 |
| 354 | Cd177 | 0.0 |
| 2820 | Themis | 0.0 |
| 2834 | Srpk3 | 0.0 |
| 2835 | Tuba3b | 0.0 |
| 367 | Aldh1a1 | 0.0 |
| 1680 | ENSRNOG00000063284 | 0.0 |
| 1681 | Kif2c | 0.0 |
| 1381 | Greb1 | 0.0 |
| 1383 | Rergl | 0.0 |
| 324 | Vtn | 0.0 |
| 329 | Dtx1 | 0.0 |
| 1366 | Nxpe2 | 0.0 |
| 1369 | Retn | 0.0 |
| 1373 | Ngfr | 0.0 |
| 1376 | Spink8 | 0.0 |
| 1684 | Nt5c1a | 0.0 |
| 1688 | Cyp2c23 | 0.0 |
| 3184 | Ttn | 0.0 |
| 1693 | Sez6l2 | 0.0 |
| 1695 | Pclo | 0.0 |
| 1350 | Adh6a | 0.0 |
| 2868 | Chrdl2 | 0.0 |
| 2869 | Cdc45 | 0.0 |
| 2873 | ENSRNOG00000066818 | 0.0 |
| 1540 | Mab21l3 | 0.0 |
| 2876 | AABR07060353.2 | 0.0 |
| 2837 | ENSRNOG00000064639 | 0.0 |
| 2838 | Mgam | 0.0 |
| 2840 | Sh2d3c | 0.0 |
| 2845 | Galnt14 | 0.0 |
| 2846 | ENSRNOG00000055586 | 0.0 |
| 937 | Acod1 | 0.0 |
| 2848 | Kcnj13 | 0.0 |
| 2850 | Kif17 | 0.0 |
| 312 | Sox18 | 0.0 |
| 832 | Pimreg | 0.0 |
| 319 | Tnmd | 0.0 |
| 1657 | Jag2 | 0.0 |
| 1659 | Bhlhe22 | 0.0 |
| 1662 | Amy1a | 0.0 |
| 834 | Gzmf | 0.0 |
| 1668 | Dgkb | 0.0 |
| 1669 | Ska1 | 0.0 |
| 1673 | Shcbp1 | 0.0 |
| 2265 | Tfap2b | 0.0 |
| 1390 | H1f1 | 0.0 |
| 1392 | Dysf | 0.0 |
| 294 | Cyyr1 | 0.0 |
| 302 | Mmp3 | 0.0 |
| 2266 | Ephb1 | 0.0 |
| 307 | Tac1 | 0.0 |
| 2242 | Tspoap1 | 0.0 |
| 2865 | Lrat | 0.0 |
| 916 | Kif4a | 0.0 |
| 921 | Pbk | 0.0 |
| 925 | Kmo | 0.0 |
| 928 | Oacyl | 0.0 |
| 929 | Ly6al | 0.0 |
| 2184 | Srl | 0.0 |
| 852 | Ntf3 | 0.0 |
| 2195 | Taar4 | 0.0 |
| 907 | Ccdc3 | 0.0 |
| 861 | Smim5 | 0.0 |
| 2881 | Itgb8 | 0.0 |
| 1998 | Bcan | 0.0 |
| 281 | Cdk1 | 0.0 |
| 1518 | Scd | 0.0 |
| 286 | Rln1 | 0.0 |
| 2885 | Scml4 | 0.0 |
| 2890 | Esrp1 | 0.0 |
| 35 | ENSRNOG00000065679 | 0.0 |
| 263 | Gata3 | 0.0 |
| 266 | Adgrl4 | 0.0 |
| 268 | Ccr6 | 0.0 |
| 271 | Klra22 | 0.0 |
| 253 | Pmfbp1 | 0.0 |
| 1531 | Ush1c | 0.0 |
| 257 | Defb14 | 0.0 |
| 2877 | ENSRNOG00000065085 | 0.0 |
| 897 | Adam18 | 0.0 |
| 2213 | Pdcd1lg2 | 0.0 |
| 3229 | Clgn | 0.0 |
| 1077 | ENSRNOG00000065917 | 0.0 |
| 3230 | Aqp3 | 0.0 |
| 946 | AABR07058479.1 | 0.0 |
| 1053 | ENSRNOG00000023005 | 0.0 |
| 1985 | ENSRNOG00000066660 | 0.0 |
| 1058 | Il2 | 0.0 |
| 1017 | Fam111a | 0.0 |
| 2916 | Fam89a | 0.0 |
| 2918 | Havcr1 | 0.0 |
| 1403 | RGD1309350 | 0.0 |
| 1407 | Exoc3l2 | 0.0 |
| 1637 | Ston1 | 0.0 |
| 1640 | Comp | 0.0 |
| 1641 | Padi4 | 0.0 |
| 1644 | AABR07007146.1 | 0.0 |
| 1675 | ENSRNOG00000063101 | 0.0 |
| 1676 | Il5 | 0.0 |
| 2888 | ENSRNOG00000038422 | 0.0 |
| 2889 | AABR07036711.1 | 0.0 |
| 1986 | Celsr1 | 0.0 |
| 1620 | Eaf2 | 0.0 |
| 1987 | Pacsin1 | 0.0 |
| 1990 | Tnfrsf13b | 0.0 |
| 2902 | Mctp1 | 0.0 |
| 2911 | Olr302 | 0.0 |
| 2912 | ENSRNOG00000027855 | 0.0 |
| 2111 | ENSRNOG00000068008 | 0.0 |
| 2113 | Mmd2 | 0.0 |
| 981 | Stum | 0.0 |
| 982 | Blk | 0.0 |
| 1066 | Nsg1 | 0.0 |
| 1072 | Cspg4 | 0.0 |
| 1074 | Cyp2s1 | 0.0 |
| 2043 | Arhgef16 | 0.0 |
| 2044 | Plxnb1 | 0.0 |
| 2953 | Tacr3 | 0.0 |
| 2954 | Vsx1 | 0.0 |
| 2956 | Scube1 | 0.0 |
| 2960 | Akap3 | 0.0 |
| 2961 | Adgrl3 | 0.0 |
| 1653 | Gbp1 | 0.0 |
| 2919 | Dpys | 0.0 |
| 2920 | Olr1058 | 0.0 |
| 2923 | Hs3st3a1 | 0.0 |
| 2930 | Mpz | 0.0 |
| 2931 | Fam189a2 | 0.0 |
| 229 | Clec4m | 0.0 |
| 233 | Siglech | 0.0 |
| 236 | Fcer1a | 0.0 |
| 237 | Selp | 0.0 |
| 1992 | Rem2 | 0.0 |
| 2973 | Klra5 | 0.0 |
| 1149 | Rasl12 | 0.0 |
| 2978 | Fgfr4 | 0.0 |
| 196 | Ms4a2 | 0.0 |
| 197 | ENSRNOG00000003209 | 0.0 |
| 1631 | Pdzrn4 | 0.0 |
| 1121 | ENSRNOG00000000871 | 0.0 |
| 1633 | Btnl9 | 0.0 |
| 866 | Grb7 | 0.0 |
| 1402 | Srms | 0.0 |
| 243 | Ly49i4 | 0.0 |
| 2244 | Fam3d | 0.0 |
| 2937 | AABR07034636.1 | 0.0 |
| 2939 | ENSRNOG00000064685 | 0.0 |
| 2950 | ENSRNOG00000037838 | 0.0 |
| 2952 | Tmem182 | 0.0 |
| 1414 | Tmem204 | 0.0 |
| 1415 | Myo5b | 0.0 |
| 1417 | Cd72 | 0.0 |
| 1420 | Erfe | 0.0 |
| 1421 | Kcnd2 | 0.0 |
| 1423 | Krt13 | 0.0 |
| 2963 | Rgs17 | 0.0 |
| 2245 | Sh3bgr | 0.0 |
| 215 | Ccl1 | 0.0 |
| 2247 | Degs2 | 0.0 |
| 226 | Spink2 | 0.0 |
| 2964 | Mb | 0.0 |
| 2965 | ENSRNOG00000062345 | 0.0 |
| 2966 | Prss3b | 0.0 |
| 2968 | Ptprv | 0.0 |
| 2971 | Sema5b | 0.0 |
| 2994 | Nphs1 | 0.0 |
| 2995 | Trdn | 0.0 |
| 2996 | Hjv | 0.0 |
| 2998 | Ms4a5 | 0.0 |
| 2999 | Olr300 | 0.0 |
| 3003 | Klhl14 | 0.0 |
| 3004 | Cd22 | 0.0 |
| 3006 | ENSRNOG00000007014 | 0.0 |
| 199 | Cyp2b1 | 0.0 |
| 200 | Map3k7cl | 0.0 |
| 1609 | ENSRNOG00000067761 | 0.0 |
| 1610 | Gpx2 | 0.0 |
| 1614 | Cdh6 | 0.0 |
| 1615 | Rasgrp2 | 0.0 |
| 1616 | RGD1560394 | 0.0 |
| 2253 | Reep1 | 0.0 |
| 183 | Cd209 | 0.0 |
| 186 | Mzb1 | 0.0 |
| 846 | Npw | 0.0 |
| 192 | Stfa2 | 0.0 |
| 193 | Il22 | 0.0 |
| 3012 | Neil3 | 0.0 |
| 3013 | Rit2 | 0.0 |
| 3020 | B3gnt7 | 0.0 |
| 3026 | Fam205a | 0.0 |
| 164 | Vsig4 | 0.0 |
| 850 | Casq2 | 0.0 |
| 204 | Adra2a | 0.0 |
| 2281 | ENSRNOG00000065916 | 0.0 |
| 2983 | Mcoln2 | 0.0 |
| 2984 | Espl1 | 0.0 |
| 2985 | RNaseP-nuc.25 | 0.0 |
| 2289 | Gjb5 | 0.0 |
| 171 | Mmrn1 | 0.0 |
| 1431 | Prdm1 | 0.0 |
| 1441 | Cpa1 | 0.0 |
| 1443 | Rbm24 | 0.0 |
| 1588 | Chodl | 0.0 |
| 1590 | ENSRNOG00000039759 | 0.0 |
| 1591 | Tlr10 | 0.0 |
| 1592 | ENSRNOG00000069978 | 0.0 |
| 1594 | Zfp683 | 0.0 |
| 1598 | ENSRNOG00000064527 | 0.0 |
| 1601 | Slamf1 | 0.0 |
| 1603 | Plk5 | 0.0 |
| 2290 | Bmx | 0.0 |
| 3011 | Fabp9 | 0.0 |
| 2291 | Dach1 | 0.0 |
| 1605 | Ube2t | 0.0 |
| 788 | Nnat | 0.0 |
| 179 | Dlk1 | 0.0 |
| 3029 | Mlph | 0.0 |
| 3031 | Palm3 | 0.0 |
| 3032 | Grxcr1 | 0.0 |
| 3033 | Arap3 | 0.0 |
| 3035 | Gpr85 | 0.0 |
| 3039 | Sirpb2 | 0.0 |
| 3047 | Slc22a2 | 0.0 |
| 3051 | Fbxo2 | 0.0 |
| 3052 | Lrrn1 | 0.0 |
| 3053 | Epha5 | 0.0 |
| 3054 | Lilra5 | 0.0 |
| 3057 | Uox | 0.0 |
| 168 | Bcam | 0.0 |
| 1428 | Nrxn1 | 0.0 |
| 154 | Fam167b | 0.0 |
| 157 | Mcpt9 | 0.0 |
| 159 | Serpine2 | 0.0 |
| 161 | Npy1r | 0.0 |
| 162 | ENSRNOG00000000167 | 0.0 |
| 795 | Srsf12 | 0.0 |
| 802 | Gimap5 | 0.0 |
| 3068 | Fam83b | 0.0 |
| 3069 | Ren | 0.0 |
| 3070 | Scg2 | 0.0 |
| 3071 | Cd209a | 0.0 |
| 3072 | Elavl4 | 0.0 |
| 3073 | Sertm1 | 0.0 |
| 3074 | Bard1 | 0.0 |
| 3075 | ENSRNOG00000062944 | 0.0 |
| 2273 | Fam131c | 0.0 |
| 1569 | AABR07006142.1 | 0.0 |
| 1466 | Ptcra | 0.0 |
| 134 | Cd79b | 0.0 |
| 139 | ENSRNOG00000000940 | 0.0 |
| 140 | ENSRNOG00000058590 | 0.0 |
| 1576 | Cd6 | 0.0 |
| 806 | A2m | 0.0 |
| 1583 | Trat1 | 0.0 |
| 1133 | Gpr15 | 0.0 |
| 1587 | Dyrk3 | 0.0 |
| 1448 | Klhl4 | 0.0 |
| 1449 | Ccr1l1 | 0.0 |
| 1136 | AABR07041403.1 | 0.0 |
| 1451 | RGD1562914 | 0.0 |
| 1453 | Agr2 | 0.0 |
| 1027 | Grem1 | 0.0 |
| 2084 | Synpo2l | 0.0 |
| 2094 | Ibsp | 0.0 |
| 997 | Rgs13 | 0.0 |
| 999 | Ly49s4 | 0.0 |
| 1000 | Umodl1 | 0.0 |
| 1003 | Diras2 | 0.0 |
| 2081 | Cyp2ab1 | 0.0 |
| 2082 | Cacna1h | 0.0 |
| 1032 | Rbpms2 | 0.0 |
| 1033 | Myb | 0.0 |
| 1040 | ENSRNOG00000011042 | 0.0 |
| 3081 | ENSRNOG00000071185 | 0.0 |
| 3089 | ENSRNOG00000064963 | 0.0 |
| 118 | ENSRNOG00000068554 | 0.0 |
| 121 | Ednrb | 0.0 |
| 3097 | Pah | 0.0 |
| 1973 | Pappa2 | 0.0 |
| 1974 | Pbld1 | 0.0 |
| 3106 | Ryr2 | 0.0 |
| 3107 | Fam181b | 0.0 |
| 772 | Olfm2 | 0.0 |
| 774 | Lrrc3b | 0.0 |
| 108 | Prf1 | 0.0 |
| 113 | ENSRNOG00000064589 | 0.0 |
| 145 | Cd69 | 0.0 |
| 775 | Fpr2 | 0.0 |
| 3076 | AABR07071622.1 | 0.0 |
| 780 | Ly49s5 | 0.0 |
| 782 | AC103574.1 | 0.0 |
| 1022 | Rtp3 | 0.0 |
| 1026 | Csta | 0.0 |
| 3146 | Klrc3.1 | 0.0 |
| 3109 | Cox6a2 | 0.0 |
| 3116 | Rflna | 0.0 |
| 3117 | Ms4a15 | 0.0 |
| 3119 | ENSRNOG00000001342 | 0.0 |
| 3120 | Prtn3 | 0.0 |
| 3124 | Ccdc88c | 0.0 |
| 3128 | Prkcq | 0.0 |
| 1545 | Troap | 0.0 |
| 786 | Mmp8 | 0.0 |
| 1553 | Slc7a15 | 0.0 |
| 1478 | RGD1311251 | 0.0 |
| 1479 | Apoc4 | 0.0 |
| 1480 | Lad1 | 0.0 |
| 1473 | Grap | 0.0 |
| 127 | Cd79a | 0.0 |
| 1525 | Scn5a | 0.0 |
| 1527 | Slc34a2 | 0.0 |
| 1530 | AABR07006032.1 | 0.0 |
| 1481 | Cdh11 | 0.0 |
| 1482 | ENSRNOG00000066444 | 0.0 |
| 1487 | Sort1 | 0.0 |
| 3133 | Syt8 | 0.0 |
| 3135 | Dbndd1 | 0.0 |
| 2308 | Lix1 | 0.0 |
| 2309 | ENSRNOG00000037476 | 0.0 |
| 2310 | Hey2 | 0.0 |
| 91 | C1qtnf9 | 0.0 |
| 92 | Cenpf | 0.0 |
| 94 | Ptprb | 0.0 |
| 99 | Emcn | 0.0 |
| 3141 | Tmem114 | 0.0 |
| 3219 | ENSRNOG00000063028 | 0.0 |
| 3236 | Kir3dl1 | 0.0 |
| 3237 | Dgke | 0.0 |
| 1059 | Scn7a | 0.0 |
| 2071 | Aqp7 | 0.0 |
| 2073 | Sv2b | 0.0 |
| 1153 | Ass1 | 0.0 |
| 2077 | Gpha2 | 0.0 |
| 3153 | ENSRNOG00000066689 | 0.0 |
| 3154 | Kcnj1 | 0.0 |
| 3155 | AABR07048770.1 | 0.0 |
| 1128 | Mxd3 | 0.0 |
| 1130 | Mlana | 0.0 |
| 817 | Ccnb2 | 0.0 |
| 1507 | ENSRNOG00000000097 | 0.0 |
| 1524 | Ovol2 | 0.0 |
| 2276 | Ankrd1 | 0.0 |
| 1539 | Rpl39l | 0.0 |
| 2148 | Lat2 | 0.0 |
| 2058 | Sgcg | 0.0 |
| 2060 | RGD1561730 | 0.0 |
| 2061 | Ptger3 | 0.0 |
| 2064 | Psrc1 | 0.0 |
| 1049 | Depdc1 | 0.0 |
| 1050 | Nkain4 | 0.0 |
| 3178 | Clec4a | 0.0 |
| 1492 | Card11 | 0.0 |
| 1494 | Havcr2 | 0.0 |
| 1503 | Avp | 0.0 |
| 1506 | Adamts18 | 0.0 |
| 1521 | Ptgds | 0.0 |
| 1537 | Cryba4 | 0.0 |
| 2152 | Tox3 | 0.0 |
| 3156 | Irf6 | 0.0 |
| 3160 | Mastl | 0.0 |
| 3168 | Tubb4a | 0.0 |
| 56 | Gzmbl2 | 0.0 |
| 2154 | Vwa2 | 0.0 |
| 3174 | Lipg | 0.0 |
| 3148 | Adcyap1r1 | 0.0 |
| 3150 | Pole2 | 0.0 |
| 3152 | Cda | 0.0 |
| 762 | Cldn1 | 0.0 |
| 769 | Cdh17 | 0.0 |
| 2332 | ENSRNOG00000065138 | 0.0 |
| 3221 | Mmp16 | 0.0 |
| 3217 | Ucn2 | 0.0 |
| 3218 | Megf10 | 0.0 |

*Note: SHAP values represent each gene contribution to the cross-study CPTC classification model. Genes with SHAP = 0 were not learned by the model and are retained to indicate non-contributing features. Index denotes the original gene ordering in the feature matrix.*
