# Supplementary material for: CPTCs Drive Somatic‐Visceral Communication via the Wnt Axis in Somatic Mechanotherapy: A Single‐Cell Deep Learning Study
Source: FASEB J. 2026 Aug 8;40(15):e72176. doi: 10.1096/fj.202600755RRR (PMC13452619; doi:10.1096/fj.202600755RRR)
Supplement: Supplementary file 3 [file FSB2-40-e72176-s007.docx]

**Table S4. Dual-Evaluation Strategy Reveals the Hierarchy of Mechanosensitive Cells (Within-Study Evaluation)**

| **Cell Type** | **Tissue** | **Acc. T (%)*** | **Acc. ST (%)*** | **Acc. G (%)*** | **G-Index*** | **MR*** |
| --- | --- | --- | --- | --- | --- | --- |
| Macrophage | Fascia | 99.34 | 99.82 | 90.07 | 0.902 | 1 |
| Fibroblast | Fascia | 99.44 | 99.91 | 86.36 | 0.864 | 2 |
| CPTCs | Fascia | 99.86 | 99.99 | 86.32 | 0.863 | 3 |
| Endothelial cell | Fascia | 96.09 | 99.15 | 85.46 | 0.862 | 4 |
| Mural cell | Fascia | 96.90 | 99.13 | 81.58 | 0.823 | 5 |
| Epithelial cell | Colon | 92.14 | 98.92 | 80.93 | 0.818 | 6 |
| Lymphocyte | Colon | 88.79 | 98.20 | 73.48 | 0.749 | 7 |
| Macrophage | Colon | 92.50 | 90.43 | 61.76 | 0.683 | 8 |
| CPTCs | Colon | 95.37 | 99.19 | 65.02 | 0.656 | 9 |
| Neuron | Colon | 89.41 | 97.85 | 58.28 | 0.596 | 10 |
| Myofibroblast | Colon | 83.10 | 96.72 | 54.87 | 0.567 | 11 |
| Fibroblast | Colon | 92.55 | 52.00 | 50.06 | 0.963 | 12 |
| Endothelial cell | Colon | 90.08 | 60.85 | 50.06 | 0.823 | 13 |
| Smooth muscle cell | Colon | 90.00 | 46.30 | 50.08 | 1.082 | 14 |
| Lymphocyte | Fascia | 94.89 | 47.62 | 57.93 | 1.216 | 15 |

*Note: G-Index (GI) = Generalization Accuracy / Self-Test Accuracy; Acc.T, Training Accuracy (%); Acc.ST, Self-Test Accuracy (%); Acc.G, Generalization Accuracy (%); MR, Mechanosensitivity Ranking.*
