# Supplementary material for: CPTCs Drive Somatic‐Visceral Communication via the Wnt Axis in Somatic Mechanotherapy: A Single‐Cell Deep Learning Study
Source: FASEB J. 2026 Aug 8;40(15):e72176. doi: 10.1096/fj.202600755RRR (PMC13452619; doi:10.1096/fj.202600755RRR)
Supplement: Supplementary file 4 [file FSB2-40-e72176-s009.docx]

**Table S2. Cross-Study G-Index Values in Forward and Reverse Transfer Directions**

| **celltype** | **fwd_gindex** | **fwd_acc** | **rev_gindex** | **rev_acc** |
| --- | --- | --- | --- | --- |
| TC | -0.0019283746556474357 | 0.4990358126721763 | 0.6051830458199985 | 0.8025915229099992 |
| FC | -0.059020044543429795 | 0.4704899777282851 | 0.43530798703212503 | 0.7176539935160625 |
| MC | -0.02275369539943528 | 0.48862315230028236 | 0.06306149149585694 | 0.5315307457479285 |
| EC | -0.31243001119820824 | 0.3437849944008959 | -0.3900709219858156 | 0.3049645390070922 |
| LP | 0.10466439135381123 | 0.5523321956769056 | -0.15786637931034486 | 0.42106681034482757 |
| MuC | -0.24395946999220575 | 0.3780202650038971 | -0.4606741573033708 | 0.2696629213483146 |

*Note: fwd, forward direction (independent cohort to current study); rev, reverse direction (current study to independent cohort). G-Index = (Accuracy - 0.5)/0.5. The reverse-direction values constitute the definitive ranking reported in Table 1.*
